# Redox regulation of PEP activity during seedling establishment in *Arabidopsis thaliana*

**DOI:** 10.1038/s41467-017-02468-2

**Published:** 2018-01-03

**Authors:** Manuel Guinea Díaz, Tamara Hernández-Verdeja, Dmitry Kremnev, Tim Crawford, Carole Dubreuil, Åsa Strand

**Affiliations:** 1 Umeå Plant Science Centre, Department of Plant Physiology, Umeå University, SE-901 87 Umeå, Sweden; 2 Present Address: Molecular Plant Biology, University of Turku, FI-20520 Turku, Finland

## Abstract

Activation of the plastid-encoded RNA polymerase is tightly controlled and involves a network of phosphorylation and, as yet unidentified, thiol-mediated events. Here, we characterize PLASTID REDOX INSENSITIVE2, a redox-regulated protein required for full PEP-driven transcription. PRIN2 dimers can be reduced into the active monomeric form by thioredoxins through reduction of a disulfide bond. Exposure to light increases the ratio between the monomeric and dimeric forms of PRIN2. Complementation of *prin2-2* with different PRIN2 protein variants demonstrates that the monomer is required for light-activated PEP-dependent transcription and that expression of the nuclear-encoded photosynthesis genes is linked to the activity of PEP. Activation of PEP during chloroplast development likely is the source of a retrograde signal that promotes nuclear *LHCB* expression. Thus, regulation of PRIN2 is the thiol-mediated mechanism required for full PEP activity, with PRIN2 monomerization via reduction by TRXs providing a mechanistic link between photosynthetic electron transport and activation of photosynthetic gene expression.

## Abstract (web summary)

The plastid-encoded RNA polymerase PEP is regulated according to plastid redox state. Here, the authors show that the redox-regulated PRIN2 protein is reduced to monomeric form in a thiol-dependent manner in response to light and that PRIN2 monomers are required for PEP activity and retrograde signaling.

## Introduction

In addition to being the primary energy source for plants, light also provides plants with information to control processes such as chloroplast development and seedling establishment^1,2^. In response to light exposure, dark-grown seedlings change the expression of approximately 30% of all nuclear-encoded genes, with genes encoding chloroplast-targeted proteins the most dramatically upregulated^3^. The establishment of photosynthesis during chloroplast development is, therefore, dependent on nuclear-encoded components^4^. However, a large proportion of the components required for photosynthesis, such as the core components of photosystem II (PSII) and PSI, and the large subunit of RUBISCO, are encoded in the plastid genome. Gene expression in the plastids is also induced by light and the activities of the plastid, and nuclear genomes must be synchronized to respond correctly to light^5–7^.

Transcription of the plastid-encoded genes of higher plants requires at least two different RNA polymerases: the nuclear-encoded plastid RNA polymerase (NEP) and the plastid-encoded RNA polymerase (PEP). NEP is a T3–T7 bacteriophage type that is mainly responsible for transcribing housekeeping genes^8^, whereas PEP is a bacterial-type multisubunit enzyme mainly responsible for transcribing photosynthesis-related genes^9^. The majority of chloroplast genes can be transcribed by either polymerase, but NEP and PEP are thought to use different promoter elements^10^. Chloroplast development is associated with a shift in the usage of the primary RNA polymerase from NEP to PEP. This process is not very well defined and the mechanism(s) behind this switch is unknown; however, in green leaves, more than 80% of all plastid genes are transcribed by PEP^11^. Similar to bacterial systems, PEP has a catalytic core composed of plastid-encoded proteins (*rpoA, rpoB, rpoC1*, and *rpoC2*)^10^. In addition to the plastid-encoded core components, the nuclear-encoded sigma factors (SIGs) are required for PEP promoter specificity^12^. Furthermore, proteomic studies have revealed that a large number of nuclear-encoded proteins are associated with the PEP core^13^. The large number of PEP-associated proteins that are potentially required for functional PEP-mediated transcription suggests that chloroplast gene expression mechanisms are complex and highly regulated^14^. However, the specific functions of these PEP-associated proteins remain unclear.

Photosynthesis strongly influences PEP-dependent plastid gene expression, and different mechanisms that link redox signals from the photosynthetic electron transport to plastid gene expression have been proposed. A complex network of phosphorylation events, directly linked to the redox status of the plastoquinone (PQ) pool, has been suggested to be involved^15–18^. It has been shown that the oxidized state of the PQ pool affects the phosphorylation state of the sigma factor SIG1, which in turn regulates the relative transcription of the photosynthesis reaction center genes *psbA* (PSII) and *psaA/B* (PSI)^19^. In addition, in organello transcription experiments performed with kinase inhibitors and the reductant dithiothreitol (DTT) suggested that a thiol-mediated signal is also involved in the redox regulation of PEP components^20^. The components of this putative thiol-mediated pathway by which the redox regulation of PEP activity occurs are currently unknown^21^, but potentially, some of the PEP-associated proteins could play a role in this regulation^14^. The PEP-associated proteins are plant-specific proteins, suggesting that those proteins evolved relatively late compared with the eubacterial core subunits of the PEP complex. Proteins that evolved late in the plant lineage can be linked to regulatory functions coordinating different processes in the cell^22^ and could potentially improve the redox regulatory system inherited from cyanobacteria^23^.

PLASTID REDOX INSENSITIVE2 (PRIN2) was identified using forward genetics as a chloroplast component involved in redox-mediated retrograde signaling^24^. The *prin2-1* and *prin2-2* alleles both demonstrated impaired regulation of photosynthesis-associated nuclear genes (*PhANGs*) when exposed to excess light, or when photosynthetic electron transport (PET)^24^ was inhibited. However, the *ys1* mutant, with impaired PEP activity, also demonstrates misregulation of *LHCB1.1* and *LHCB2.4* expression in response to excess light^24^ that is similar to the *prin2* mutants, suggesting that changes to PEP activity, rather than PRIN2, specifically, is the source of redox-mediated retrograde signal(s). PRIN2 is, however, required for correct expression of plastid-encoded photosynthesis genes^24^. Plastid transcriptome analyses show that PRIN2 is essential for full expression of genes assigned to be PEP dependent, both in *Arabidopsis* seedlings and rosette plants^24^. In addition, *PhANG* expression levels were low compared to WT in *prin2* seedlings grown under control conditions. GENOMES UNCOUPLED 1 (GUN1) was shown to be genetically linked to PRIN2 because the impaired expression of *PhANGs* under control conditions was reverted to near-wild-type levels in the *prin2/gun1-1* double mutant^24^. Here, we demonstrate a direct interaction between PRIN2 and a component of the PEP complex, Thioredoxin z (TRXz). We also show that PRIN2 can be regulated via thioredoxins (TRXs). Our results indicate that a reduced monomeric form of the PRIN2 protein is the active form required for light-activated PEP-dependent transcription in the plastids. In response to the establishment of electron transport activity during seedling establishment and possibly during leaf development, a thioredoxin-like protein generates a switch from dimer to monomeric form of PRIN2. Full activation of the PEP complex and plastid photosynthetic gene expression is achieved, and as a consequence also full expression of the nuclear-encoded photosynthesis genes.

## Results

### PRIN2 is required for plastid transcriptional activity

**Fig. 1:**
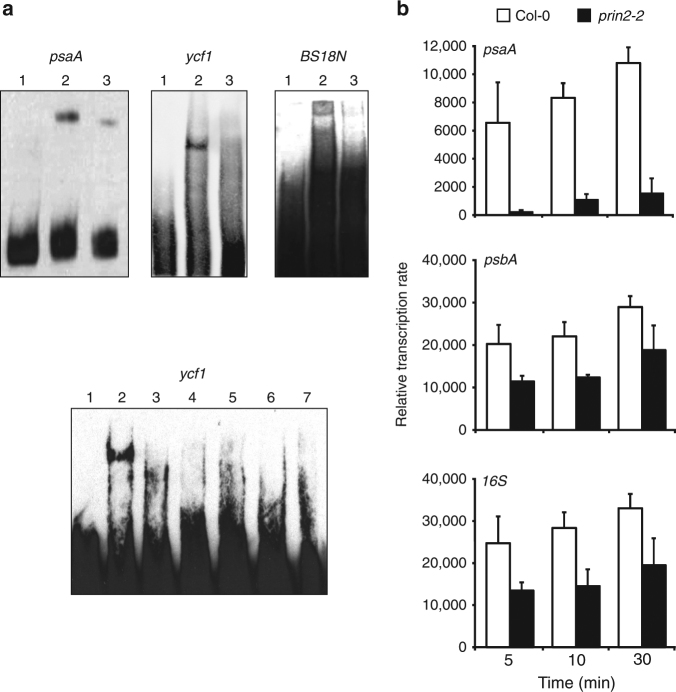
The role of PRIN2 in transcriptional activity. **a** DNA binding of PRIN2 to DNA fragments in EMSA assays. Binding of PRIN2 to PEP promoter-containing probe *psaA*, NEP promoter-containing probe *ycf1*, and oligonucleotide BS18N, which contained random 18mer sequences^26^. Lane 1 free probe, lane 2 probe + PRIN2, and lane 3 competition analysis, probe + PRIN2 + unlabeled probe. Competition using PEP and NEP promoter-containing probes for PRIN2/*ycf1* complex binding in EMSA assays. Lane 1 free ycf1, lane 2 *ycf1* + PRIN2, and lanes 3–7 *ycf1* + PRIN2 + unlabeled probe corresponding to *ycf1* (3), *rpoB* (4), *clpP* (5), *psaA* U region (6), or *psbA* (7). Competition was done with 50-fold excess unlabeled probe over the labeled probe. A total of 3 μg of purified protein was used for every reaction. Two independent experiments were performed. **b** Run-on transcription assay in Col-0 and *prin2-2* isolated chloroplasts. Filters with *psaA*, *psbA,* and *16S* probes, were hybridized with total RNA from Col-0 and *prin2-2* isolated chloroplasts. Data shown are the mean ± s.d. from quantification of three independent blots and experiments

PRIN2 was previously shown to have DNA-binding capacity, both alone and in a complex with the conserved chloroplast protein, chloroplast stem-loop binding protein 41 kDa (CSP41b)^25^. To investigate the possibility for sequence-specific DNA binding, recombinant PRIN2 protein was used to perform electrophoretic mobility shift assay (EMSA) with promoter fragments of the PEP-dependent *psaA* and NEP-dependent *ycf1*. DNA/PRIN2 complexes were observed for both DNA fragments tested in the EMSA (Fig. 1a), confirming the DNA-binding capacity of PRIN2, but suggesting that the DNA interaction is unspecific. To further test this general DNA-binding ability of PRIN2, EMSAs were performed with a random DNA primer probe, BS18N^26^, and nonlabeled DNA fragments corresponding to PEP or NEP promoters as competitors of *ycf1*/PRIN2 binding (Fig. 1a). The binding to the random DNA primer probe, and the ability of all the nonlabeled DNA fragments to compete for interaction with the labeled *ycf1* DNA probe strongly indicates that the interaction between PRIN2 and DNA sequence is nonspecific, implying that PRIN2 is not involved in the recognition of the promoters like the PEP-associated sigma factors^12^.

Plastid transcriptome analyses of *prin2* mutant alleles clearly demonstrated that the expression levels of genes assigned to be PEP-dependent genes were significantly lower in *prin2* compared to wild type (WT), whereas expression of the plastid housekeeping genes was higher compared to WT^24^. Numerous publications have demonstrated that mutants with impaired PEP activity, all show expression profiles similar to *prin2*, high transcript levels of NEP-dependent genes, and low levels of PEP-dependent genes compared to WT^27–30^. Thus, given the consistency of the responses of these other mutants, the plastid gene expression profile suggested that PRIN2 is required for full expression of genes transcribed by PEP^24^. To investigate if the decreased steady-state levels of the PEP-dependent transcripts observed in the *prin2* mutants^24^ were due to decreased transcriptional activity rather than posttranscriptional effects such as decreased RNA stability, we performed a run-on transcription assay for *psaA*, *psbA*, and *16S rRNA*, all assigned to be PEP-dependent transcripts, in wild- type and *prin2-2* mutant chloroplasts. This assay demonstrated that the levels of newly synthesized transcripts were significantly lower in the *prin2-2* mutant compared to WT for *psaA*, *psbA*, and *16S rRNA* (Fig. 1b). Taken together, the results suggest that PRIN2 is required for transcriptional activity rather than influencing RNA stability.

### PRIN2 is a redox-regulated protein

**Fig. 2:**
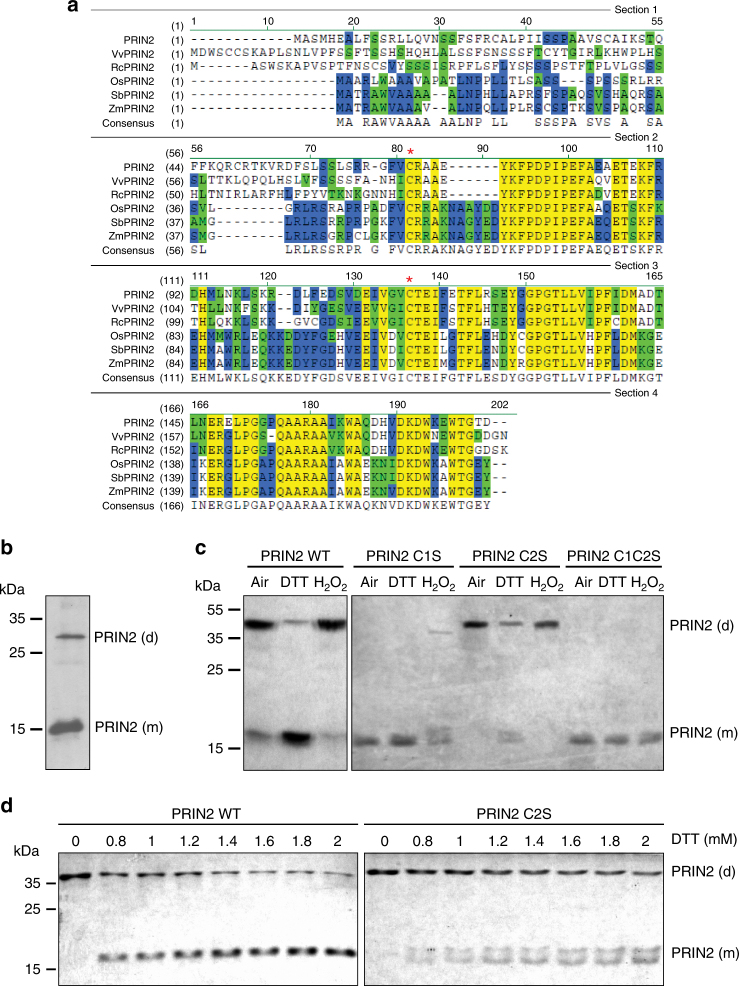
Conserved redox-responsive cysteine residues identified for PRIN2. **a** The amino acid sequences of PRIN2 proteins from *Arabidopsis thaliana*, *Vitis vinifera* (Vv), *Ricinus communis* (Rc), *Oryza sativa* (Os), *Sorghum bicolor* (Sc), and *Zea mays* (Zm), were aligned and manually adjusted. Identical residues are shadowed in yellow, 50% identical residues in blue, and similar residues in green. Red asterisks mark the conserved cysteines. **b** Immunoblot analysis of PRIN2 with 100 μg of protein from isolated chloroplasts of a 35 S:PRIN2 line under nonreducing conditions. **c** Protein electrophoresis of recombinant *Arabidopsis* PRIN2 WT and the mutated versions PRIN2 Cys68Ser (C1S), PRIN2 Cys115Ser (C2S), and PRIN2 Cys68SerCys115Ser (C1C2S). Purified recombinant proteins (1 μg) were treated with 5 mM DTT, 2 mM of H_2_O_2_, or untreated (air); and subjected to SDS-PAGE (15% polyacryalmide) under nonreducing conditions. **d** Protein electrophoresis of recombinant PRIN2 WT and PRIN2 C2S. Purified recombinant protein (1 μg) was treated with different DTT concentrations and subjected to SDS-PAGE (15% polyacrylamide) under nonreducing conditions. **c**, **d** Gels were stained with Coomassie Brilliant Blue. **b**–**d** Molecular mass markers (kDa) are shown on the left and at least two independent experiments were performed

PRIN2 is absent from the genomes of green algae and cyanobacteria, indicating that this protein appeared during evolution of the plant lineage and suggesting that it might perform a regulatory function^22^. Analysis of the PRIN2 protein sequences from different plant species showed that the protein lacked putative kinase or phosphatase domains, but revealed the presence of two conserved cysteine residues within the mature protein, Cys68, and Cys115 (Fig. 2a). Cys68 is located at the N terminus and Cys115 in the middle of the mature PRIN2 protein. The strict conservation of cysteines between homologous proteins could be used as a guide to identify reactive cysteines^31^. Thus, the two conserved cysteine residues suggested a potential for redox regulation, and posttranslational modifications. Previous work showed that PRIN2 can exist in the form of monomers, dimers, and oligomers^25^, and the presence of two conserved cysteine residues suggests the possibility for redox regulation via intermolecular disulfide bonds. To test this hypothesis, western blots were performed using protein extracts from isolated chloroplasts of a 35S:PRIN2 line subjected to SDS-PAGE under nonreducing conditions. The western blot showed two bands, corresponding to PRIN2 monomer and dimer, and the dominating form under normal light-grown conditions is the monomer (Fig. 2b). To confirm the two forms of the PRIN2 proteins, recombinant PRIN2 protein was expressed and subjected to nonreducing SDS-PAGE. Again, two bands were observed, corresponding to the monomer and the dimer (Fig. 2c). Dimer formation was induced during oxidative conditions when H_2_O_2_ was added. In contrast, reducing conditions attained by the addition of DTT, resulted in an accumulation of the monomeric form (Fig. 2c). To investigate the specific involvement of the Cys68 and Cys115 residues in this redox-regulated change, we constructed mutated variants of the PRIN2 protein where the Cys68 or Cys115 residues were substituted for Ser, termed as PRIN2 C1S and PRIN2 C2S, respectively. We also made a PRIN2 variant with both cysteines mutated, termed as Cys68Ser/Cys115Ser (PRIN2 C1C2S). The response of the mutant variants of the PRIN2 protein was analyzed in vitro. Following nonreducing SDS-PAGE, the PRIN2 C1S mutant protein was present only in the monomeric form irrespective of the DTT or H_2_O_2_ treatments, while the PRIN2 C2S mutant variant could change between the two forms in response to the redox conditions, suggesting that Cys68 is responsible for the dimer formation of PRIN2 and that Cys115 is not a redox-active cysteine (Fig. 2c). However, the reduction of the Cys68–Cys68 disulphide bond in the PRIN2 C2S protein variant required higher concentrations of DTT compared to what was required to bring the wild-type protein into the monomeric form (Fig. 2d). This suggests that mutating Cys115 might affect the redox activity and/or availability of Cys68 to redox regulation. Taken together, these data indicate that PRIN2 responds to changes in the redox environment and that Cys68 is required for PRIN2 redox-dependent dimer formation in vitro.

### Interaction between PRIN2 and PAPs

**Fig. 3:**
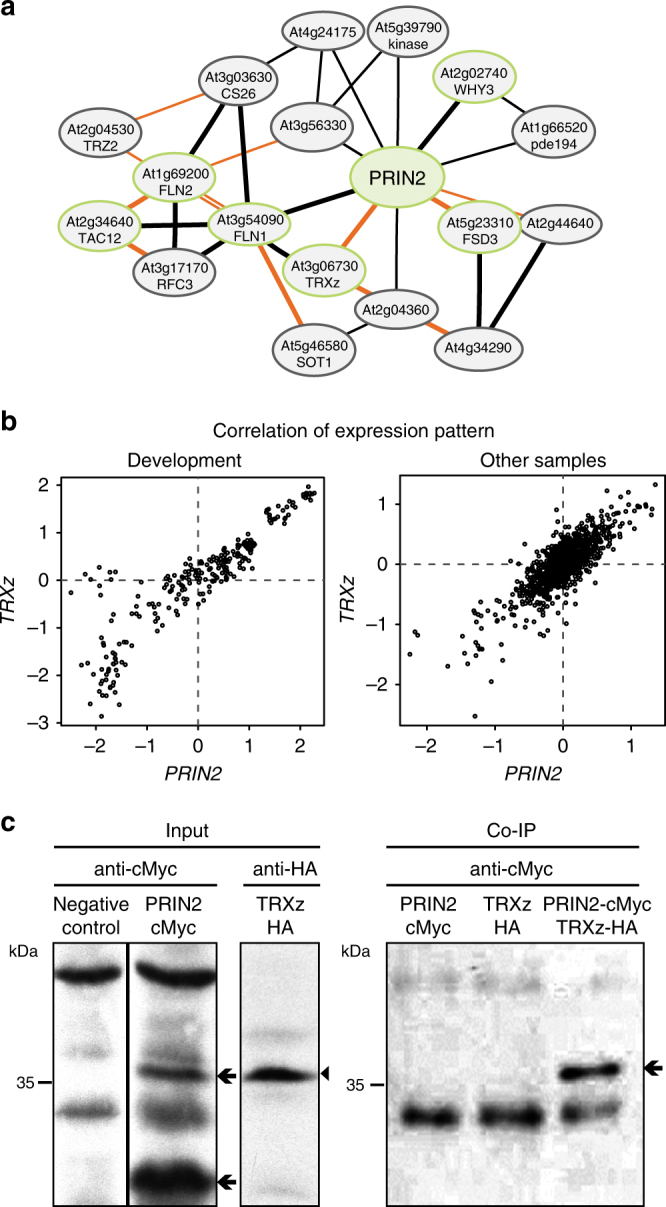
Interaction of PRIN2 with PAPs. **a** Gene coexpression network. The network was generated using PRIN2 as query. Thicker lines between nodes correspond to stronger coexpression between the linked genes. Orange lines between nodes indicate highly reliable coexpression supported by data from different data sets. Nodes corresponding to genes encoding chloroplast nucleoid proteins are outlined in green. **b** Correlation of expression pattern for PRIN2 and TRXz. Contribution of each experimental data to PRIN2 and TRXz expression pattern similarity is displayed. The samples are divided in “development” and “others” to avoid masking effects on the expression pattern due to dynamic expression changes in different tissues and growth stages (development). **a**, **b** The analyses were performed using ATTED-II v8.0^32^. **c** Co-immunoprecipitation of PRIN2 and TRXz. Input lines show immunoblots performed with protein extracts from nontransformed protoplasts (negative control), protoplasts transformed with PRIN2-cMyc or TRXz-HA, and probed with anti-cMyc or anti-HA antibodies. Co-IP was performed with *Arabidopsis* protoplasts expressing one or both PRIN2-cMyc and TRXz-HA proteins. TRXz-HA was immunoprecipitated using magnetic beads linked to anti-HA antibodies, and the Co-IP signal was detected by immunoblot probed with anti-cMyc antibody. Two independent experiments were performed. Arrows indicate PRIN2 protein and arrowhead TRXz, and molecular mass markers (kDa) are shown on the left

PRIN2 was shown to be located to the plastid nucleoids^24^ and to be required for full transcriptional activity of PEP (Fig. 1b). Coexpression analysis using ATTED-II v.8.0^32^ revealed that *PRIN2* forms a network enriched in genes encoding plastid nucleoid proteins, and several coexpressed genes encode polymerase-associated proteins (PAPs), suggesting that expression of these genes is coordinated and that the proteins may functionally interact (Fig. 3a, Supplementary Data 1). Interestingly, a significant correlation of gene expression was found for PRIN2 and TRXz (Fig. 3b). TRXz was identified as a component of the transcriptionally active chromosome (TAC)^33^ and one of the ten PAPs confirmed to be tightly associated with the PEP core. Published work indicated that changes of the redox state during light/dark transitions of fructokinase-like protein 2 (FLN2) were mediated by TRXz^33^. Thus, given the redox regulation of PRIN2 (Fig. 2), TRXz was selected as a putative interacting partner. To investigate in vivo if PRIN2 and TRXz directly interact with each other, we transiently expressed the full-length PRIN2 and TRXz fused to cMyc- and HA tags, respectively, in *Arabidopsis* protoplasts and performed a co-immunoprecipitation (Co-IP). In the PRIN2-cMyc-transformed protoplast, two specific bands of approximately 20 and 40 kDa were observed, corresponding to the PRIN2 monomer and dimer, respectively (Fig. 3c). In the TRXz-HA-transformed protoplasts, one band of approximately 40 kDa was observed, corresponding to a dimer of TRXz (Fig. 3c). After the immunoprecipitation with anti-HA and detection with anti-cMyc antibody, PRIN2 was detected in the Co-IP fraction of protoplasts transformed with both TRXz-HA and PRIN2-cMyc, demonstrating an interaction between TRXz and PRIN2 (Fig. 3c). PRIN2 physically interacts with TRXz, and is thus associated with the PEP core.

### PRIN2 monomerization is triggered by light

Following exposure of dark-grown seedlings to light, *PRIN2* expression was rapidly induced (Supplementary Fig. 1a). However, when *PRIN*2 expression levels were determined during seedling development, the levels of expression declined with age of the seedlings (Supplementary Fig. 1b). Also, when the expression profile was investigated in an *Arabidopsis* cell culture, where the greening process was carefully controlled^34^, *PRIN2* expression was very strongly induced by light but declined with time of light exposure (Supplementary Fig. 1c). The expression profile of *PRIN2* is similar to what was observed for the *SIG* factors and other PEP-associated proteins, suggesting an important role for PRIN2 in the nuclear control over chloroplast development during the early light response^34^.

**Fig. 4:**
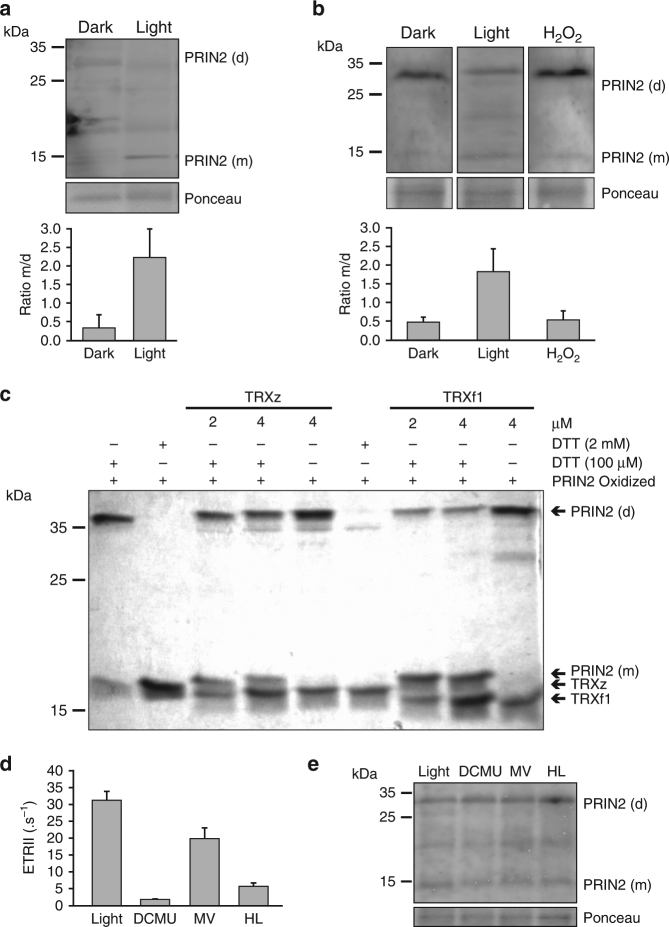
PRIN2 monomer and dimer formation. **a** Immunoblot analysis of PRIN2 from isolated chloroplasts from *Arabidopsis* under nonreducing conditions. Chloroplasts were isolated from 14-day-old plants, exposed to the dark for 48 h or maintained in light. **b** Immunoblot analysis of PRIN2 from *Arabidopsis* cell cultures. Samples were collected from dark-grown cells, cells grown in light, and cells grown in light and incubated with H_2_O_2_ (10 mM) for 3 h. **a**, **b** The graphs show the mean ratio ± s.d. between the monomer and dimer from four and three independent immunoblots and experiments, for **a** and **b**, respectively. Quantification of protein levels was done using the program ImageJ. **c** Protein electrophoresis analysis of PRIN2 with recombinant PRIN2 oxidized with H_2_O_2_ (2 mM), dialyzed, and submitted to different treatments with TRXz, TRXf1, and/or DTT. A total of 1 μg of the treated PRIN2 was subjected to SDS-PAGE (15% polyacrylamide) under nonreducing conditions. Gels were stained with Commassie Brilliant Blue. Monomer (m) and dimer **d** PRIN2 forms, TRXz and TRXf1 are shown in the figure with arrows. **d** Estimation of electron transport rate of PSII (ETRII in electrons per second) at 208 s following incubation with DCMU (50 μM), MV (200 μM), or exposure to high light (1000 -μmol photons m^−2^ s^−1^) for 3 h. Data shown are the mean ETRII parameter ± s.e.m. for three replicates and are representative of two independent biological experiments. **e** Immunoblot analysis of PRIN2 from *Arabidopsis* cell culture from the treatments in **d**. **a**, **b**, **e** Immunoblot analyses were performed with 40 μg of protein. Membranes were stained with Ponceau for loading control. Representative blots from three independent experiments are shown. **a**–**c**, **e** Molecular mass markers (kDa) are on the left

We have demonstrated both in vivo (Fig. 2b) and in vitro (Fig. 2c) that PRIN2 exists in dimeric and monomeric forms, and that changes in the redox environment can trigger an exchange between the two forms in vitro (Fig. 2). To investigate whether the shift between the two forms of PRIN2 is linked to the activity of photosynthetic electron transport, we performed in vivo experiments where the distribution between monomeric and dimeric forms of PRIN2 was compared in protein extract from isolated chloroplasts from WT *Arabidopsis*. Prior to the chloroplast isolation, the plants were exposed to a prolonged dark period and, as a comparison, to normal growth conditions in light. In the samples from the dark-treated *Arabidopsis* plants, the dimer is the dominant form, whereas in light, the monomer dominates (Fig. 4a, Supplementary Fig. 2). In addition, proteins were extracted from the *Arabidopsis* cell culture^34^ grown either in dark, normal light (150 μmol photons m^−2^ s^−1^), or treated with H_2_O_2_ (10 mM) in light. When the cells were grown in light, the distribution between the two PRIN2 forms is shifted toward the monomer (Fig. 4b). This is similar to what was observed in the chloroplast samples from *Arabidopsis* plants (Fig. 4a). When the cells were treated with H_2_O_2_, a significantly higher amount of the dimer compared to the monomer was found (Fig. 4b). Thus, incubation with H_2_O_2_ triggered a shift toward the PRIN2 dimer, suggesting that H_2_O_2_ treatment leads to oxidation of Cys68 as was observed in vitro (Fig. 2c).

The shift from dimeric to monomeric PRIN2, mediated via reduction of the Cys68–Cys68 disulphide bond, appears to be linked to light and photosynthetic activity (Fig. 4a). Over 20 TRX and TRX-like proteins have been described in *Arabidopsis* chloroplasts^35^. These TRXs can be reduced by two redox systems: the ferredoxin-thioredoxin reductase system (FTR) associated with PSI that reduces the dithiol groups of TRXs in response to the activity of the photosynthetic electron transport chain in a light-dependent manner, and the recently identified NADPH-dependent thioredoxin reductase C (NTRC) that is dependent on NADPH and can also function under dark conditions^36–38^. We analyzed whether chloroplast thioredoxins (TRXs) could reduce PRIN2 and modify the levels of the dimeric and monomeric forms of the protein in vitro. PRIN2 was shown to interact with TRXz (Fig. 3c), so, we expressed and purified TRXz and incubated it together with the PRIN2 protein. The PRIN2 monomer accumulated when incubated with TRXz in the presence of a reductant (Fig. 4c), indicating that TRXz could reduce PRIN2. However, TRXz has been assigned different biochemical properties compared to other plastid TRXs^39–41^. TRXf1 was shown to be an effective regulator of chloroplast enzymes and to be reduced by the FTR system^39^. We therefore also tested whether TRXf1 could reduce PRIN2. TRXf1 could reduce PRIN2 in the presence of a reductant and the reduction efficiency was higher compared to what was observed for TRXz (Fig. 4c). The reduction of PRIN2 by TRXs further supports the redox regulation of PRIN2 via the formation of disulphide bonds, and suggests that chloroplast TRXs may serve as potential reducing factors modulating the dimeric/monomeric forms of PRIN2 in response to light via FTR and functional PET.

We have demonstrated that monomerization of PRIN2 is light dependent (Fig. 4a, Supplementary Fig. 2) and that in light, PRIN2 is reduced, and the monomer is formed by TRXs (Fig. 4c). To test whether PRIN2 alternates between the monomeric and dimeric forms in response to fluctuations in the redox state of the photosynthetic electron transport chain, we incubated light-adapted cells with methyl viologen (MV), which blocks the flow of electrons from PSI to FTR (Fig. 4d, Supplementary Fig. 3). Following treatment with MV, no interchange between the PRIN2 forms was observed (Fig. 4e), indicating that the maintenance of the PRIN2 monomer is not dependent on a constant flow of electrons from PSI-FTR. Second, to test whether the monomerization of PRIN2 could be linked to another component of the photosynthetic electron transport, such as the PQ pool which would still be reduced following the MV treatment, we incubated light-adapted cells with 3-3,4-dichlorophenyl-1,1-dimethylurea (DCMU), which blocks the flow of electrons from PSII to PQ, leaving PQ and everything downstream in the electron transport chain oxidized (Fig. 4d, Supplementary Fig. 3). Similar to the incubation with MV, the DCMU treatment did not trigger an interchange between the two forms of PRIN2 (Fig. 4e). In addition, we exposed the cells to photoinhibitory high light conditions (1000 μmol photons m^−2^ s^−1^) and following this short-term high light treatment, we also observed no significant change in distribution between the forms (Fig. 4e). Taken together, these data show that PRIN2 does not rapidly cycle between the dimeric and monomeric forms in response to fast changes to the redox status of the photosynthetic electron transport chain. Thus, PRIN2 is not a redox sensor per se. Our results rather suggest that PRIN2 is converted to the monomeric form in response to the establishment of photosynthetic electron transport through the FTR–TRX system.

### PRIN2 monomer is required for plastid transcription

**Fig. 5:**
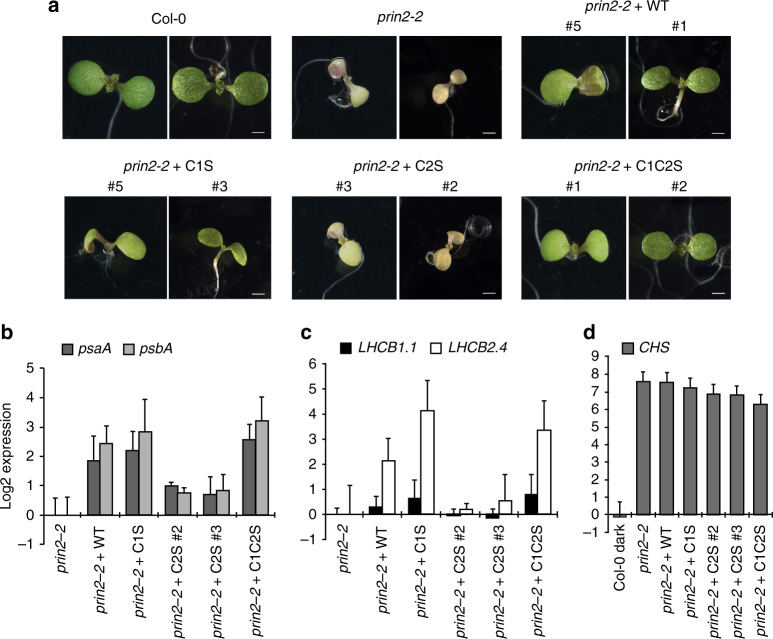
Developmental and molecular phenotypes of *prin2-2* complemented with different PRIN2 protein variants. **a** Representative images of 7-day-old seedlings of Col-0, and *prin2-2*, transformed with PRIN2 WT, PRIN2 C1S, PRIN2 C2S, and PRIN2 C1C2S. Bars = 0.1 cm. **b**, **c** Relative log2 expression levels of **b** chloroplast-encoded photosynthetic genes *psaA* and *psbA*, **c** nuclear-encoded photosynthetic genes *LHCB1.1* and *LHCB2.4*, and **d** light- inducible *CHS* in 7-day-old *prin2-2* and *prin2-2* complemented with PRIN2 WT, PRIN2 C1S, PRIN2 C2S, and PRIN2 C1C2S seedlings. Gene expression was normalized to *AT4G36800* and related to the amount present in *prin2-2* or Col-0 in the dark. Each data point represents the mean ± s.d. of at least three biological replicates

To test whether the exchange between the monomer and dimer of PRIN2 is essential for PRIN2 function, PEP activity, and expression of plastid-encoded photosynthesis genes *in planta*, we transformed the *prin2-2* null mutant allele^24^ with constructs carrying WT, Cys68Ser (C1S), Cys115Ser (C2S), or Cys68Ser/Cys115Ser (C1C2S) variants of the PRIN2 protein. Transgenic lines with *PRIN2* expression levels similar to what was found in WT were selected for further analysis (Supplementary Fig. 4a) and the presence of the PRIN2 protein in the transgenic lines was confirmed by western blot (Supplementary Fig. 4b). The pale phenotype of the *prin2-2* seedlings^24^ was complemented in independent transgenic lines transformed with the PRIN2 WT, C1S, and C1C2S variants of the PRIN2 protein (Fig. 5a). In contrast, the independent lines transformed with C2S did not complement the phenotype and thus the pale phenotype was maintained in these plants (Fig. 5a).

The *prin2-2* mutant displayed significantly lower expression levels of the PEP-dependent genes in seedlings^24^, thus, we investigated the expression levels of *psaA* and *psbA* in the transgenic lines with the different versions of PRIN2. Consistent with the recovery of the pale phenotype of *prin2-2*, the PRIN2 WT, C1S, and C1C2S plants also recovered *psaA* and *psbA* expression in 7-day-old seedlings (Fig. 5b), while the plants transformed with the C2S variant did not recover *psaA* nor *psbA* expression (Fig. 5b). In addition, even if not statistically significant, the lines transformed with C1S and C1C2S variants showed slightly higher expression levels of *psaA* and *psbA* compared to the line transformed with the wild-type variant of PRIN2 (Fig. 5b) although similar levels of the PRIN2 protein were present in the C1S and C1C2S lines compared to the wild-type variant (Supplementary Fig. 4b).

In addition to the reduced transcription of PEP-dependent genes, both *prin2* mutant alleles showed repressed *LHCB* expression compared to wild-type seedlings under control conditions^24^. This effect of the *prin2* mutation on *LHCB* expression in seedlings is consistent with the known effects of inhibitors of plastid transcription and translation that blocks the induction of expression of genes encoding light-harvesting complex (LHC) and the small subunit of Rubisco (RBCS)^42–44^. In the transgenic lines, the expression levels of the nuclear-encoded photosynthesis genes, *LHCB1.1* and *LHCB2.4*, mirrored the expression levels of the plastid-encoded *psaA* and *psbA* genes (Fig. 5c). While the WT, C1S, and the C1C2S variants of PRIN2 recovered *LHCB1.1* and *LHCB2.4* expression, the C2S variant did not (Fig. 5c). Expression of the light-induced gene *CHALCONE SYNTHASE* (*CHS*) shown to be independent of chloroplast status^45^, was however unaffected in the *prin2-2* mutant and the transgenic lines (Fig. 5d), demonstrating that other aspects of light signaling are normal in these mutants.

Neither the pale phenotype, the impaired PEP activity, nor the *LHCB* expression was recovered in the line transformed with the C2S variant of the PRIN2 protein (Fig. 5). The inability of the C2S variant to recover the *prin2-2* phenotype could be explained by the in vitro behavior of the protein variant that clearly required higher concentrations of DTT to reduce the Cys68–Cys68 bond and to generate the monomer compared to the WT variant of the PRIN2 protein (Fig. 2d). In addition, the dimeric form of PRIN2 is clearly the dominating form in the transgenic *prin2-2* + C2S lines (Supplementary Fig. 4c). The importance of the monomeric PRIN2 form during light activation of PEP was further supported when the *prin2-2* line was transformed with a Cys115Ser variant of PRIN2 with a C-terminal 4xMyc tag (C2S-4xMyc). In these plants, the PRIN2 C2S-4xMyc protein can only be detected in the monomeric form, and the developmental and molecular phenotype was also fully complemented (Supplementary Fig. 5), suggesting that the large 4xMyc tag allosterically interfered with the formation of the dimer.

### PRIN2 monomer activates the PEP complex in response to light

**Fig. 6:**
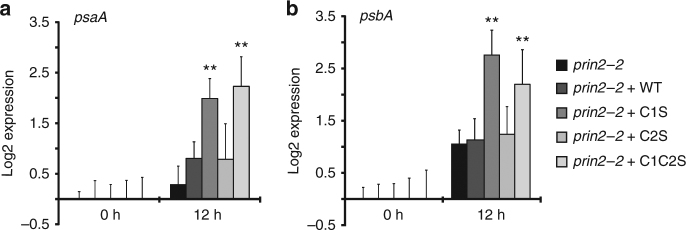
Photosynthetic gene expression during de-etiolation in *prin2-2* and complemented with PRIN2 variants. Relative log2 expression levels of PEP- transcribed *psaA* **a** and *psbA* **b** genes in *prin2-2* complemented with PRIN2 WT, PRIN2 C1S, PRIN2 C2S, and PRIN2 C1C2S seedlings 0 and 12 h after de-etiolation. Gene expression was normalized to *AT4G36800* and related to the amount present in the dark. Each data point represents the mean ± s.d. of at least three biological replicates. The *prin2-2* C1S, *prin2-2* C2S, and *prin2-2* C1C2S lines were compared to *prin2-2* WT. Significant differences were calculated with a Student´s *t*-test (**P = 0.01)

The results from the transgenic lines strongly suggested that the monomeric form of PRIN2 is required for its activity and possibly for PEP-driven transcription. According to this model, the transition from dimer to monomer in response to light and photosynthetic electron transport activity could contribute to the light-regulated control of PEP activity. During the de-etiolation process, transmission electron microscopy of the ultrastructure of plastids showed that the chloroplasts have a well-developed structure with numerous grana and intergranal thylakoids following 12 h of light exposure (Supplementary Fig. 6, Supplementary methods). Photosynthetic activity could also be observed at this time point (Supplementary Fig. 7). After 12 h of light exposure, photosynthetic electron transport is active, the PET components reduced, as shown by the qL parameter (Supplementary Fig. 7, Supplementary methods), and thus are able to generate a reduced plastid FTR/TRX system that in turn could reduce PRIN2. In addition, the expression levels of the plastid-encoded photosynthesis genes *psaA, psaC, psbA*, and *psbD* demonstrated a clear threshold between 12 and 24 h of light exposure when expression accelerated, suggesting that the shift in major RNA polymerase usage, from NEP to PEP usage occurs at this time point (Supplementary Fig. 8). If the active monomeric form of PRIN2 is generated in response to the establishment of photosynthetic electron transport, and if PRIN2 is one component of the light-induced regulation of PEP activity, the transgenic lines with PRIN2 locked in the monomeric form (C1S and C1C2S) should respond faster to light. To test this hypothesis, a de-etiolation assay was carried out using the transgenic lines, and the expression of *psaA* and *psbA* was investigated in dark-grown seedlings following 12 -h exposure to light (Fig. 6). The lines complemented with the C1S and C1C2S variants of PRIN2 showed a faster increase of *psaA* and *psbA* expression levels in response to light compared to the line transformed with PRIN2 WT or C2S (Fig. 6), supporting the role of PRIN2 as a component of the light-induced regulation of PEP-mediated transcription.

## Discussion

**Fig. 7:**
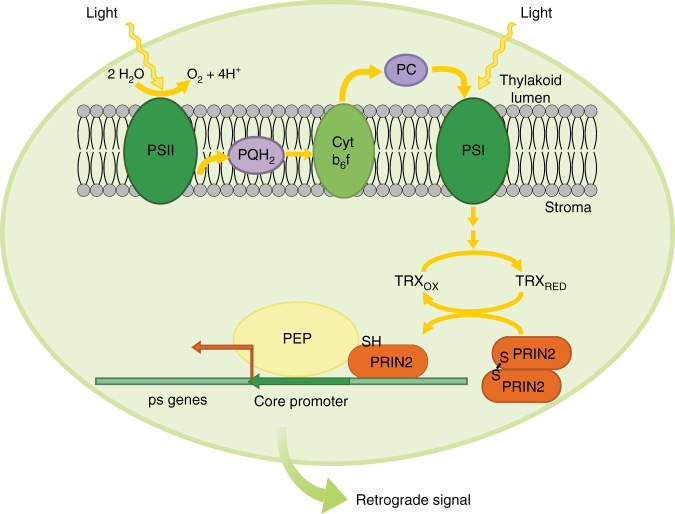
Working model for redox regulation of PRIN2 and PEP activity. A working model depicting the redox regulation of PRIN2 required for full activation of the PEP complex in the plastids. During the greening process, photosynthetic activity is gradually established and once photosynthetic electron transport (PET) is activated, the reduction of the PET components triggers reduction of TRX via the FTR system. In response to the redox changes triggered by light and photosynthetic activity, TRX converts the PRIN2 dimer into the monomer through a disulfide bond reduction. The monomeric form is the active form that promotes full initiation of light-activated transcription of plastid-encoded photosynthesis genes (ps genes). In response to full PEP activity, a retrograde signal is produced, a signal that is required for full expression of the nuclear-encoded photosynthesis genes during seedling establishment. Thus, the activities of the two genomes are tightly linked to each other. PEP, plastid-encoded RNA polymerase, PRIN2 PLASTID REDOX INSENSITIVE 2, TRX thioredoxin

Chloroplast gene expression is a complex and highly regulated process, and the mechanistic link between photosynthetic electron transport activity and regulation of plastid gene expression has been pursued for many years^46^. A major target of photosynthetic redox signals is the PEP complex^20^, although the molecular details behind this redox regulation have so far been elusive. Here, we present a model for thiol-mediated regulation of PEP-dependent transcription based on redox regulation of PRIN2 activity. During the greening process, the photosynthetic electron transport chain is established and once its components are reduced by light, the FTR/TRX system is activated. TRX converts the PRIN2 dimer into the active monomeric form through reduction of the Cys68–Cys68 disulfide bond. The monomeric form boosts light-activated transcription of photosynthetic genes in the chloroplast. As a consequence, the activation of the PEP complex generates a positive retrograde signal that regulates expression of the nuclear-encoded photosynthesis genes synchronizing the activities of the two genomes in response to light (Fig. 7).

The development of functional chloroplasts and the establishment of photosynthesis is an intricate process involving several cellular compartments. First, chloroplast development is dependent on light and the initial light signal triggers activation of the photoreceptors, initiating large changes in nuclear transcription^1,47^. Second, expression of the plastid-encoded photosynthesis genes needs to be initiated and this induction depends upon the expression and assembly of the nuclear-encoded components required for the activity of PEP^14^. Gel filtration and mass spectrometry analysis from different plant species lead to the conclusion that the transcriptionally active complex in chloroplasts contains 43 nuclear-encoded proteins^14^. Thus, given its complex structure, activation of PEP is most likely a rate-limiting step for initiation of photosynthetic gene expression in the plastids and it was therefore suggested to represent a developmental bottleneck in the establishment of photosynthesis^48^. Additionally, activation of PEP also includes redox control mechanisms that influence plastid gene expression^21^.

PRIN2 is a plant-specific protein with DNA-binding activity (Fig. 1a)^25^ with no homologous proteins found in prokaryotic, green algae, or cyanobacterial organisms^24^. We demonstrate coexpression between *PRIN2* and several of the characterized *PAP* genes (Fig. 3a, b). Furthermore, PRIN2 was shown to colocalize with PTAC12 to the plastid nucleoids^24^ and to interact with one of the well-defined PAP proteins, TRXz (Fig. 3c). PRIN2 is required for full transcriptional activity of PEP (Fig. 1b), but is not involved in target specificity (Fig. 1a), suggesting that an interaction with other protein(s) could be responsible for the sequence-specific interaction required for transcription. The PRIN2 amino acid sequence contains domains with high homology to the transactivation domain (TAD) described in yeast and mammalian systems (Supplementary Fig. 9). The nine-amino-acid, 9aaTAD, defines a TAD common to a super family of eukaryotic transcription factors and TAD was shown to promote transcription by mediating interaction between transcription factors and coactivators^49,50^. Although the plastids have prokaryotic origin and plastid transcription is thus different to the nucleus, it is possible that PRIN2 helps recruiting PEP-associated proteins promoting full activation of the PEP complex via a regulatory domain such as the 9aaTAD.

Two cysteine residues in the PRIN2 sequences are strictly conserved in homologous proteins of different plant species (Fig. 2a). In vitro, and more importantly in vivo, experiments showed that PRIN2 dimerization responds to redox regulation (Figs. 2 c, d, and 4), and that the dimer is formed through a disulfide bond between Cys68 of the PRIN2 monomers (Fig. 2c). The second conserved cysteine (Cys115) does not seem to be involved in dimer formation (Fig. 2c) but replacing Cys115 with Ser, suggested an important structural role. In the Cys115Ser variant of the protein, the dimer was more stable and very difficult to reduce (Fig. 2d, Supplementary Fig. 4c). The importance of the PRIN2 monomer for PRIN2 function was demonstrated by the *prin2-2* complementation experiments. Recovery of the morphological and molecular phenotypes was demonstrated in the lines complemented with the WT PRIN2 and the PRIN2 variants that only exist in the monomeric form, Cys68Ser (C1S), Cys68Ser/Cys115Ser (C1SC2S), and Cys115Ser with a C-terminal 4xMyc tag (C2S-4xMyc), but not with the Cys115Ser (C2S) version of the protein (Fig. 5a–c).

Four main routes of redox regulation exist in chloroplasts: (1) ferredoxin (Fd), (2) NADPH, (3) thioredoxin (TRX) system, and (4) glutathione/glutaredoxin^51^. The PRIN2 monomer accumulated when incubated with both TRXz and TRXf1 in the presence of a reductant (Fig. 4c). Although the plastid TRXf1 was shown to be an efficient reductant of the PRIN2 dimer in vitro (Fig. 4e), due to the lack of phenotype of the *trxf1* mutant^52^, we assume that other plastid TRXs or the NTRC system are involved in vivo. TRXs are reduced by the ferredoxin-thioredoxin reductase system (FTR) in response to activity of the photosynthetic electron transport chain^51^. Thus, the reduction of PRIN2 by a TRX links formation of the PRIN2 monomer and full activation of the PEP complex to the establishment of photosynthetic activity during seedling development. Following exposure to light, the monomer is clearly the dominant PRIN2 form in *Arabidopsis* plants (Fig. 4a). During the greening process, a well-developed chloroplast structure with numerous grana and intergranal thylakoids was observed following 12 h of light exposure (Supplementary Fig. 6). In addition, significant photosynthetic activity was demonstrated by chlorophyll fluorescence measurements (Supplementary Fig. 7). This is a critical time point since it coincides with a clear threshold for PEP-driven transcription. A significant increase in the expression levels for plastid-encoded photosynthesis genes was observed between 12 and 24 h of light exposure, suggesting that PEP becomes the major RNA polymerase at this time point (Supplementary Fig. 8). Thus, our model suggests that in response to initiation of photosynthetic electron transport activity, PRIN2 is activated via the FTR system and full activation of PEP-driven transcription is established. This is supported by the observation that the plants with PRIN2 locked in the monomeric form (C1S and C1C2S) responded faster in terms of induction of *psaA* and *psbA* expression, in response to light compared to the lines complemented with the wild-type version of the PRIN2 protein (Fig. 6). However, in contrast to the switch from dark to light conditions, inhibition of photosynthetic electron transport did not result in any interchange between the two forms of PRIN2 (Fig. 4e), suggesting that PRIN2 undergoes the dimer-to-monomer transition, specifically in response to activation of the FTR system. Short-term inhibition of photosynthesis using inhibitors or photoinhibitory light did not revert the monomer to the dimer as was observed following a direct treatment with H_2_O_2_ (Fig. 4b). Thus, the results indicate that once the monomer is formed, it is stable, and PRIN2 does not play a role as a redox sensor, although it is dependent for its activation on redox reactions of the photosynthetic electron transport.

Expression of nuclear-encoded photosynthesis genes was also impaired in the *prin2-2* mutant (Fig. 5c), but as PEP activity was restored in the PRIN2-complemented lines, the expression of *LHCB* was also restored (Fig. 5c). Thus, there is a clear link between photosynthetic gene expression in the plastids and the nucleus. The coordination of the activities of the two genomes is mediated by a retrograde signal. We have previously shown that the *gun1* mutation rescues the impaired expression of *PhANGs* in *prin2-2* during seedling establishment^24^. The nature of the retrograde signal is unknown, but it has been suggested that blocked chloroplast development would either disrupt a positive plastid-emitted signal, which acts in a GUN1-regulated manner, or induce a negative plastid-emitted signal, which acts to repress nuclear transcription in a GUN1-mediated manner^53,54^. Recent data describing the establishment of photosynthesis during chloroplast development suggest that full *PhANG* induction is dependent on a positive signal from healthy developing plastids^34^. In addition, here, we show that *LHCB* expression is directly correlated with the recovery of PEP activity in the different *prin2*-complemented lines (Fig. 5c), suggesting that it is a positive signal generated by PEP activity in the plastids that stimulates *LHCB* expression in the nucleus. Taken together, the results suggest that the monomeric form of PRIN2 is the active form of the protein, and contributes to the activation of the PEP complex during the establishment of functional chloroplasts in response to light. Once PEP is activated, a positive retrograde signal is triggered and thus, the status of the PEP complex links the functional state of the chloroplast to the nucleus, enabling the plant to synchronize expression of photosynthetic genes from the nuclear and chloroplast genomes during seedling development.

## Methods

### Plant material and growth conditions

*Arabidopsis thaliana* Columbia-0 (Col-0) was used for all the experiments. The *Arabidopsis thaliana* T-DNA insertion line *prin2-2* (GK-772D07-024643) was previously described^24^. Seedlings were grown on plates containing 1 × Murashige and Skoog (MS) salt mixture supplemented with vitamins (Duchefa M0222), 1% sucrose, and Phytagel (Sigma P8169). Seedlings and rosette plants were grown on soil at 23 °C (16 -h light 100-μmol photons m^−2^ s^−1^) and 18 °C (8 h in the dark). For de-etiolation assays, seeds were plated on MS 1% sucrose, stratified, exposed for 3 h to light, and grown in the dark for 5 days. De-etiolation was induced in constant white light.

Chloroplasts isolation was performed using a two-step Percoll (Sigma P7828) gradient^55^ with 8 g of tissue from 14-day-old plants. A pluripotent inducible cell line was generated from *Arabidopsis* Col-0^34^. This habituated cell line can be propagated in the dark in MS supplemented with 3% sucrose without the addition of hormones or growth factors. In these cells, chloroplast development can be triggered on demand by exposing the culture to light and reducing sucrose to 1%^34^. For the experiments, the cells were cultured in MS with 1% sucrose, and shifted to continuous light. After 7 days in light, the cultures were incubated in control conditions with no addition, mixed with 10 mM H_2_O_2_, 50 µM 3-(3,4-dichlorophenyl)-1,1-dimethylurea (DCMU) or 200 µM methyl viologen (MV), or shifted to high light (1000- μmol photons m^−2^ s^−1^) to man-ipulate the redox state of the photosynthetic electron transport chain. Cells were incubated in either control or inhibitory conditions for 3 h before taking samples for protein extraction and measurements of photosynthetic activity.

### Cloning and directed site mutagenesis of Cys residues

For *prin2-2* mutant transformation, a PCR fragment of 540 bp containing the complete coding sequence of *PRIN2* was amplified from Col-0 cDNA with the primers listed in Supplementary Table 1, and was subcloned in pDONR207 (Invitrogen) to obtain pENTRY PRIN2. For the 4xMyc-tagged versions, we amplified 791 bp of the promoter region and the genomic sequence of PRIN2 with the primers listed in Supplementary Table 1, and subcloned it in pDONR207 (Invitrogen) to obtain the pENTRY PRIN2-Myc. The different variants of PRIN2 protein were obtained by directed site mutagenesis using the pENTRY PRIN2 or pENTRY PRIN2-Myc as template and the primers listed in Supplementary Table 1. All the variants obtained were cloned into the binary vector pH2GW7^56^ or pGWB16^57^ for the fusion to 4xMyc. The plants were transformed using the floral-dip method^58^. Overall, the following independent lines were scored for the reported phenotype for each construct, the *prin2-2* transformed with pH2GW7, PRIN2: *prin2-2 + PRIN2 WT*, six independent lines:  *prin2-2 + PRIN2 C1S*, three independent lines: *prin2-2 + PRIN2 C2S*, three independent lines: *prin2-2 + PRIN2 C1C2S*, and two independent lines. The *prin2-2* transformed with pGWB16, PRIN2-4xMyc: *prin2-2 + PRIN2 WT-4Myc*, seven independent lines: *prin2-2 + PRIN2 C1S-4Myc*, four independent lines: *prin2-2 + PRIN2 C2S4-Myc*, seven independent lines: *prin2-2 + PRIN2 C1C2S-4Myc*, and 11 independent lines.

For the expression of recombinant proteins, the coding sequence of PRIN2 and TRXz without the transit peptide was amplified using cDNA as a template and the primers listed in Supplementary Table 1. PCR products were cloned into NcoI-AccI sites in pET-His1a vector (kindly provided by Günter Stier, Umeå University, Sweden). The vector to overexpress TRXf1 was kindly provided by Francisco Javier Cejudo, University of Seville, Spain. The vectors were transformed in BL21 *Escherichia coli* cells.

### cDNA synthesis and real-time PCR

Total RNA was isolated using Plant RNA Mini Kit (EZNA) and genomic DNA was removed by DNase I treatment (Thermo-Fisher EN0525). cDNA was synthesized with iScript cDNA Synthesis kit (Bio-Rad 1708891) according to the manufacturer’s instructions and 10× diluted. Real-time PCR was performed using iQSYBR Green Supermix (Bio-Rad 1725006CUST) in a CFX96 Real-Time system (C1000 Thermal Cycler; Bio-Rad) with a two-step protocol. The primers used are listed in Supplementary Table 1. All experiments were performed with three biological and three technical replicates, and relative gene expression was normalized to the expression of *AT4G36800*. Data analysis was done using CFX manager (Bio-Rad) and LinRegPCR software^59^.

### Plastid run-on transcription assay

Run-on transcription assay^60^ was performed with isolated chloroplasts^55^ of Col-0 and *prin2-2* seedlings. The *psaA*, *psbA*, and *16SrRNA* DNA probes were linked to a Hybond-N + membrane (Amersham RPN3050B) and hybridized with total RNA extract from the respective chloroplasts. The signal was revealed by overnight exposure to the X-ray film at –80°C. Quantification was done with ImageJ software.

### EMSA

Double-stranded oligonucleotide *BS18N*, which contained random 18mer sequences flanked by BamHI, EcoRI, SalI, and XhoI sites, was prepared by annealing oligonucleotides bs-18N and rs-1 listed in Supplementary Table 1, followed by primer extension with Klenow fragment^26^. DNA probes for the promoter regions of PEP- and NEP-transcribed genes were PCR amplified using the primers listed in Supplementary Table 1, and 3′-end labeled with Biotin-14-dCTP (Invitrogen 19518018) using Biotin 3′ End DNA Labeling kit (Thermo Fisher 89818) according to the manufacturer’s instructions. DNA was UV cross-linked to the membrane, incubated with streptavidin-HRP, and detected with Chemiluminescence Nucleic Acid Detection Module (Thermo Fisher 89880) according to the manufacturer’s instructions.

### Co-immunoprecipitation

Full-length PRIN2 and TRXz coding sequences were amplified using primers listed in Supplementary Table 1 and cloned into NcoI site in pRT104-Myc and into SacI–SacII in pRT104-3HA vectors. *Arabidopsis* L*er* suspension culture cells were used to prepare protoplast, and the protoplast was transiently transformed with pRT104-PRIN2-cMyc and pRT104-TRXz-HA using PEG transformation protocol^61^. Co-IP was performed using anti-HA antibody (Covance MMS-101R) bound to protein G-coated magnetic beads. The binding of the antibody, the immunoprecipitation, and the elution were performed according to the manufacturer's instructions (Dynabeads Protein G Immunoprecipitation Kit, Invitrogen 10007D). Input and Co-IP samples were subjected to western blot and detected with anti-cMyc chicken IgY (1:1000, Thermo Fisher A-21281) and rabbit HRP-linked anti-chicken IgY (1:20000, Sigma A9046), or HRP-linked anti-HA (1:1000, Sigma 12013819001).

### Thioredoxin assay

The PRIN2, TRXf, and TRXz proteins were overexpressed in *E. coli*, after induction with 1 mM IPTG for 6 h, and purified by NTA chromatography in Tris-HCl Buffer (Tris-HCl, 20 mM pH 7.9, 500 mM NaCl, and 10% glycerol). The elution of the proteins was induced adding the same buffer with 500 mM imidazole to the column. For the in vitro reduction, recombinant PRIN2 was previously oxidized by a 5- min treatment with 2 mM H_2_O_2_. Then, the protein was dialyzed using Centrifugal Filter Units (Millipore) in the same buffer described previously. Two micromolar of oxidized PRIN2 were incubated in the presence of TRXf1 or TRXz (2 or 4 µM), with or without DTT (100 µM) for 30 min at 25 °C. In the positive control, 2 mM DTT was used as a reductant. After incubation, 1 µg of treated PRIN2 was loaded in a 15% acrylamide gel and subjected to SDS-PAGE under nonreducing conditions. Gels were stained with Coomassie Brilliant Blue.

### Protein gel electrophoresis and immunoblotting

Total proteins were extracted with extraction buffer, 10% SDS, 20% glycerol, 0.2 M Tris, pH 6.8, 0.05% Bromophenol blue, and with 5% β-mercaptoethanol when indicated, separated on 15% acrylamide SDS-PAGE, and transferred to 0.45 -µm nitrocellulose membrane (Amersham 10600003). Proteins were detected with rabbit anti-PRIN2 polyclonal antibody (1:200, Agrisera) or mouse c-myc (9E10) monoclonal antibody (1:10000, Covance MMS-150R), and secondary HRP-linked anti-rabbit (1:10000, Agrisera AS09 602) or HRP-linked anti-mouse (1:10000, Amersham NA931) antibodies. Luminescence was detected using ECL Prime Western Blotting Detection Reagent (Amersham RPN2232) and LAS-3000 Imaging System (Fuji). Quantification of the monomer and dimers of PRIN2 was done with ImageJ software, using the loading control levels for each point to normalize each data point. The specificity of the PRIN2 antibody is shown in Supplementary Fig. 10. The full uncropped versions of the blots are shown in Supplementary Fig. 11.

### Data availability

The authors declare that all data supporting the findings of this study are included in the main manuscript file or Supplementary Information or are available from the corresponding author upon request.

## Electronic supplementary material

Supplementary Information

Description of Additional Supplementary Files

Supplementary Data 1
