# Supplementary material for: Redox regulation of PEP activity during seedling establishment in Arabidopsis thaliana
Source: Nat Commun. 2018 Jan 3;9:50. doi: 10.1038/s41467-017-02468-2 (PMC5752674; doi:10.1038/s41467-017-02468-2)
Supplement: Supplementary file 1 — Supplementary Information [file 41467_2017_2468_MOESM1_ESM.pdf]

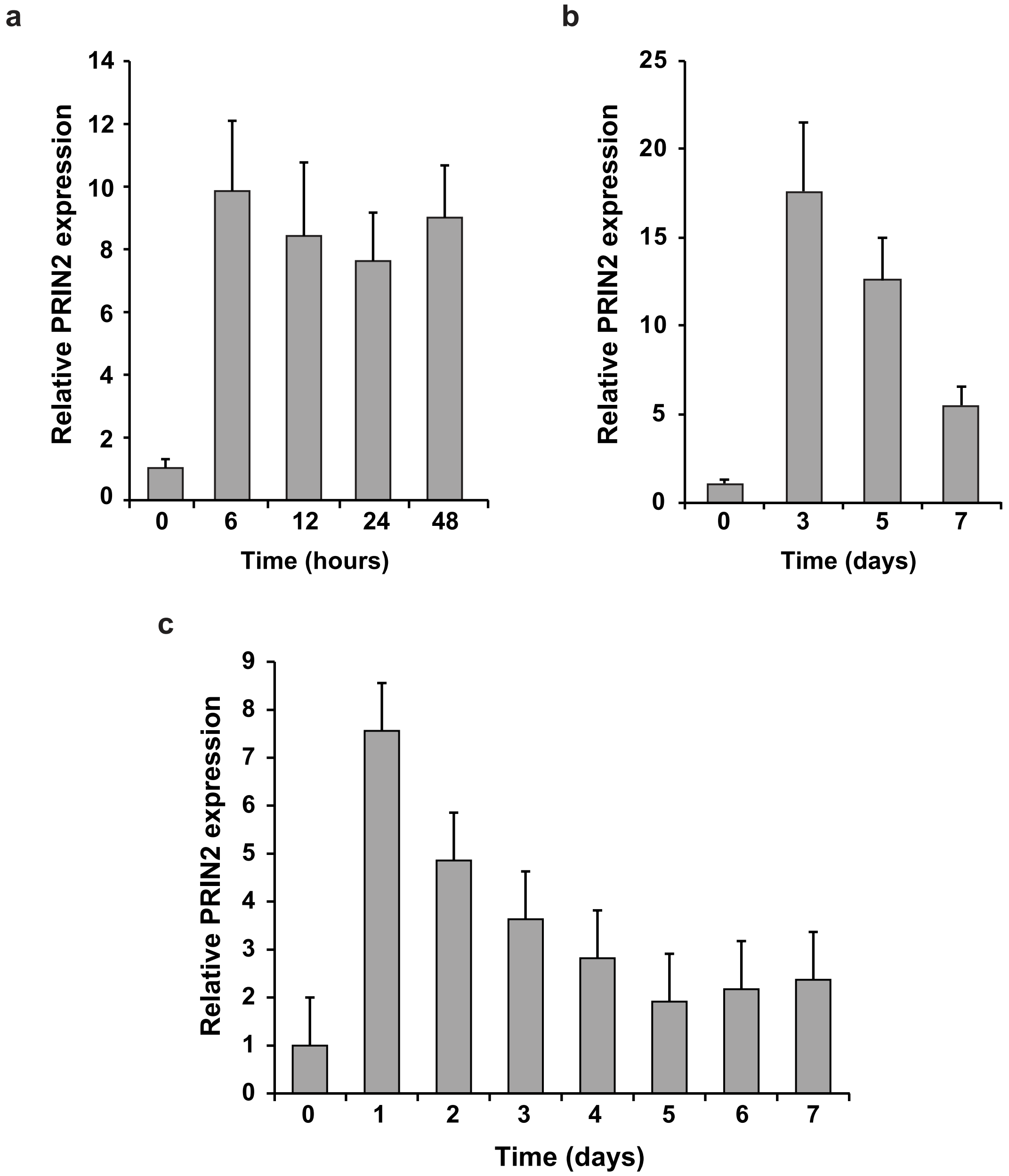

Supplementary Figure 1. Relative *PRIN2* expression in Arabidopsis

Relative expression of *PRIN2* in (**a**) 5-day-old dark grown etiolated Arabidopsis WT seedlings shifted to light, (**b**) WT seedlings grown in light, and in (**c**) WT Arabidopsis cell culture at different times following exposure to light. Gene expression was normalized to *AT4G36800* and related to the amount present at time 0. Data represents the mean ± s.d. of at least three independent biological replicates.

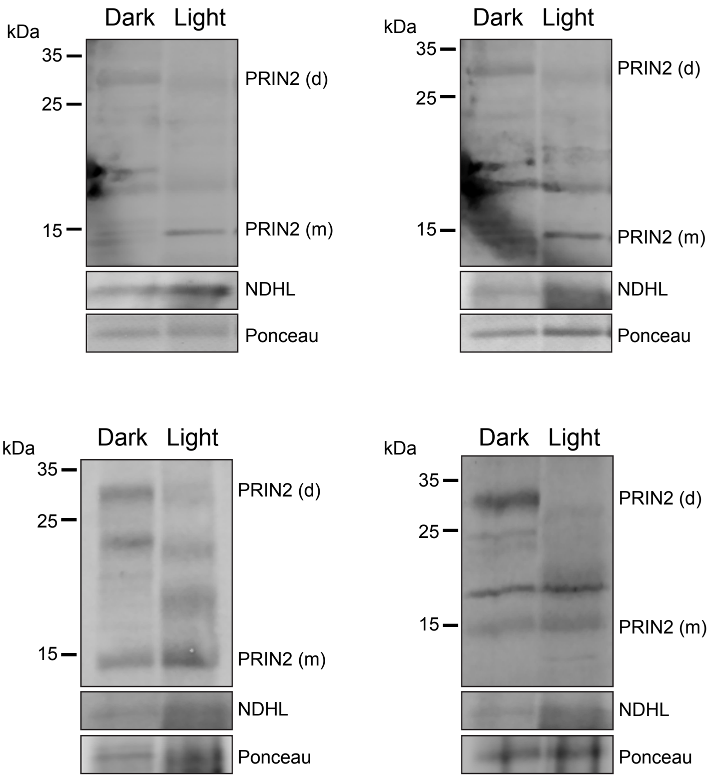

Supplementary Figure 2. Immunoblot analyses of PRIN2 from isolated chloroplast
Independent immunoblots of PRIN2 from Arabidopsis isolated chloroplast under non-reducing conditions. Chloroplasts were isolated from 14-day-old plants, exposed to dark 48h or maintained in the light. Immunoblots were performed using 40 μg of protein. Membranes were probed with specific anti NDHL antibody[1] and stained with Ponceau for loading control. Molecular mass markers (kDa) are shown in the left.

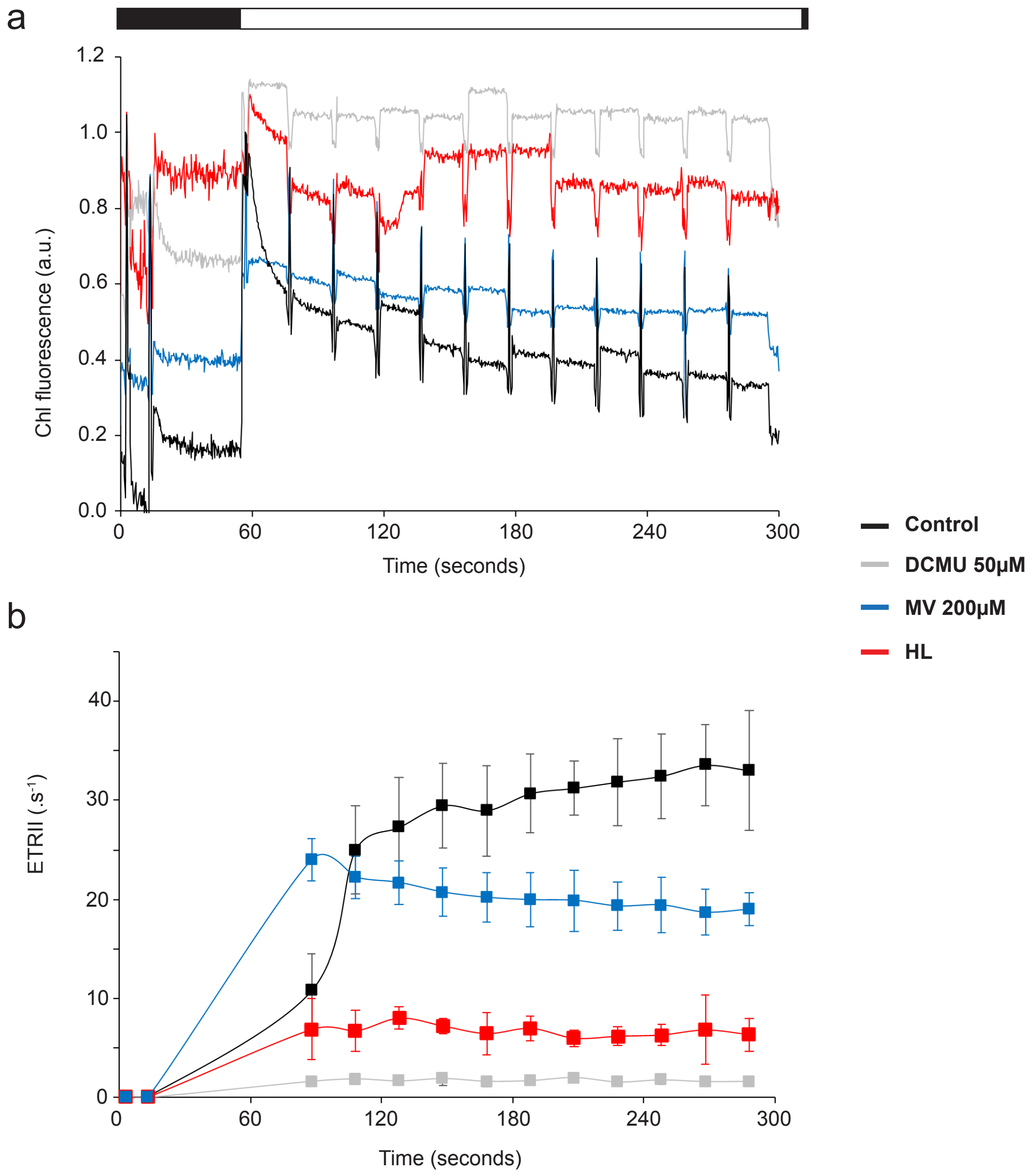

Supplementary Figure 3. Manipulation of the photosynthetic electron transport

(**a**) Kinetics of chlorophyll *a* fluorescence and (**b**) estimation of electron transport rate of PSII centres (ETRII in electrons per second) in 7-day-old WT Arabidopsis cell culture grown in light and incubated in control conditions (black) or treated with 50 µM DCMU (grey), 200 µM MV (blue) or 1000 µmol photons m^{-2} s^{-1} white light (red) for 3 h. Application of 100 µmol photons m^{-2} s^{-1} red actinic light is indicated by the white bar, and dark periods by the black bars. (**a**) The average kinetic traces from 3 technical replicates are shown normalized to Fm'. (**b**) Data shown are the mean ETRII parameter ± s.e.m. for 3 replicates and are representative of 2 independent experiments.

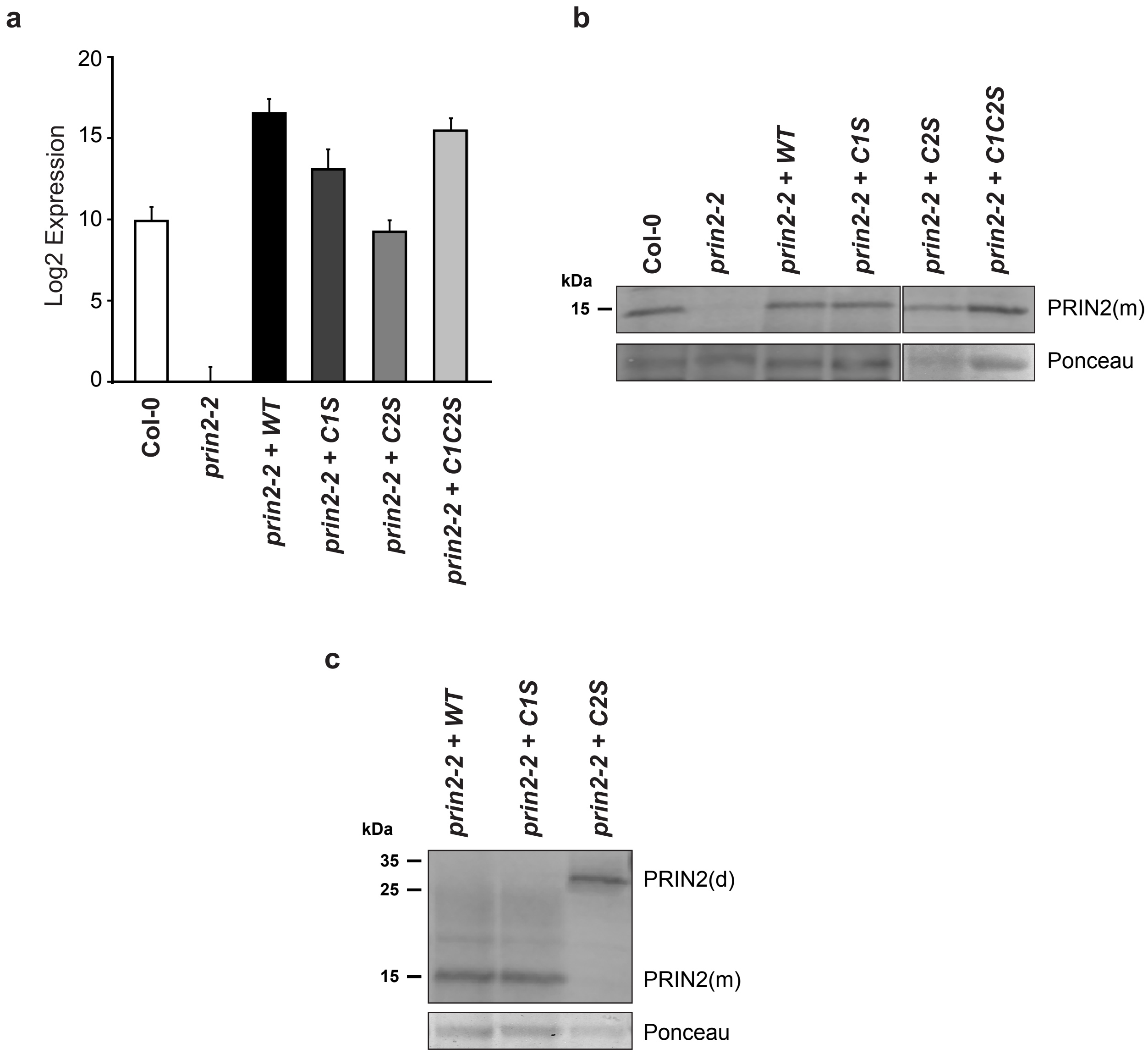

Supplementary Figure 4. Relative *PRIN2* expression and PRIN2 protein levels

(**a**) Relative log2 expression levels in 7-day-old Arabidopsis seedlings of *PRIN2* in WT, *prin2-2,* and the transgenic *prin2-2* lines transformed with the different PRIN2 variants. Gene expression was normalized to *AT4G36800* and related to the amount present in *prin2-2*. Data represents the mean ± s.d. of three independent biological replicates. (**b**) PRIN2 monomer levels. Immunoblot analysis of PRIN2 with 70 µg of protein from isolated chloroplasts from WT, *prin2-2,* and transgenic *prin2-2* lines transformed with the different PRIN2 variants. Proteins were subjected to SDS-PAGE (15% polyacrylamide) under highly reducing conditions (100 mM DTT) and blotted. (**c**) It was difficult to reduce the C2S variant of the PRIN2 protein to the monomer as shown by this immunoblot analysis with 70 µg of protein from isolated chloroplasts from the transgenic *prin2-2* lines transformed with the WT, C1S, or C2S PRIN2 variants. Proteins were subjected to SDS-PAGE (15% polyacrylamide) under reducing conditions and blotted. (**b-c**) Representative blots from 3 independent experiments are shown. Molecular mass markers (kDa) are shown on the left. Membranes were stained with Ponceau for loading control.

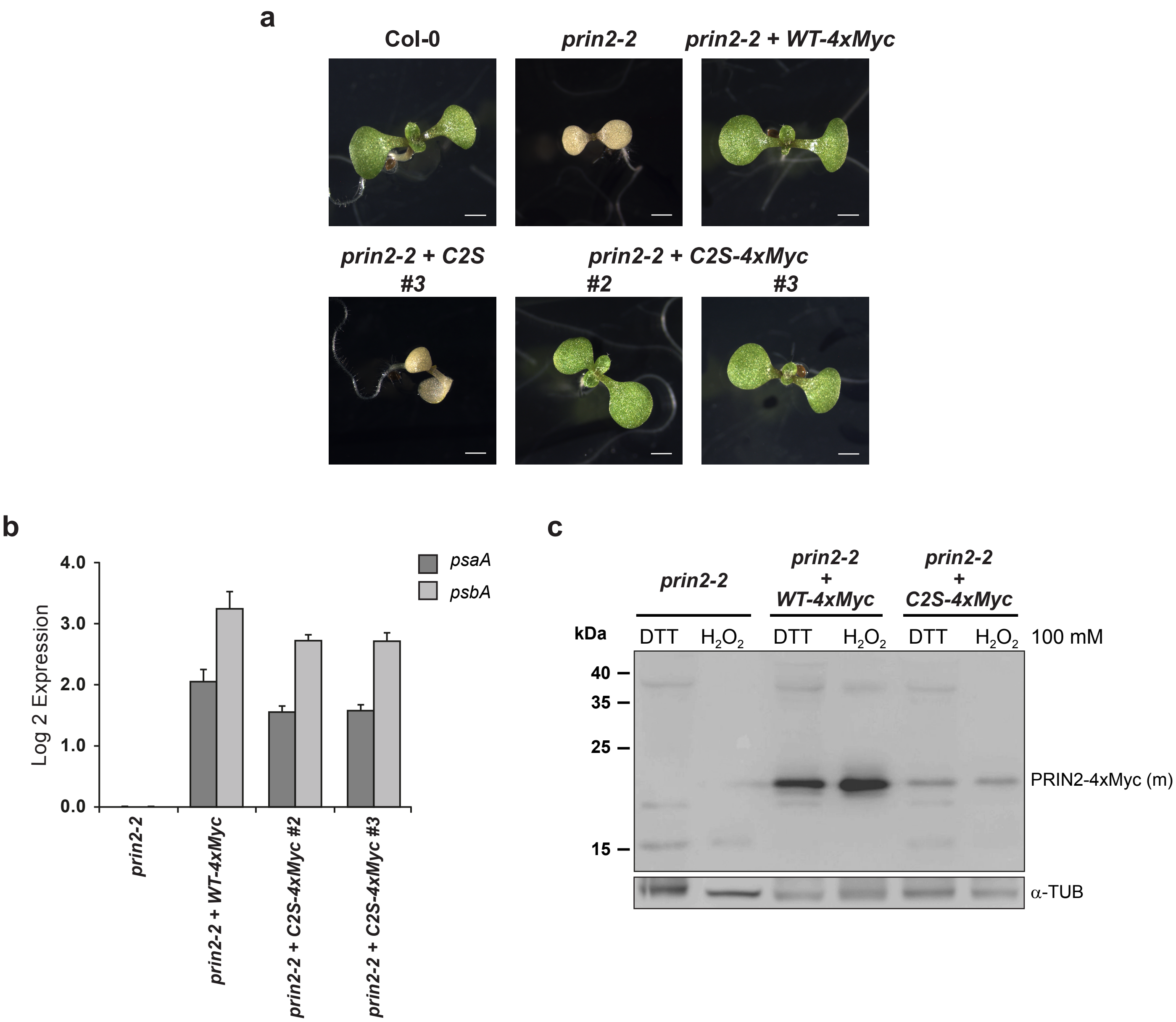

Supplementary Figure 5. Developmental and molecular phenotypes of *prin2-2* transformed with PRIN2 protein variants tagged with 4xMyc epitope.

(**a**) Representative images of 7-day-old Arabidopsis seedlings of Col-0, *prin2-2*, *prin2-2* transformed with PRIN2 WT fused to 4xMyc (*prin2-2* + WT-4xMyc), and 2 independent lines of PRIN2 C2S fused to 4xMyc (*prin2-2* + C2S-4xMyc). Bars = 0.1cm. (**b**) Relative log2 expression levels of chloroplasts encoded photosynthetic genes *psaA* and *psbA* in 7-day-old seedlings of *prin2-2* and *prin2-2* transformed with PRIN2 WT-4xMyc, and 2 independent lines of PRIN2 C2S-4xMyc. Gene expression was normalized to *AT4G36800* and related to the amount present in *prin2-2*. Data represents the mean ± s.d. of three independent biological replicates. (**c**) Immunoblot analysis of PRIN2-4xMyc with 50 μg of protein from isolated chloroplasts from *prin2-2*, *prin2-2* WT-4xMyc, and *prin2-2* C2S-4xMyc. Proteins were treated with DTT (100mM) or H_2O_2 (100 mM) and were subjected to SDS-PAGE (15% polyacrylamide) under non reducing conditions and blotted. Unspecific interaction with the anti-Myc antibody appears in the *prin2-2* mutant background. An antibody against α-Tubulin was used as loading control. Representative blot from 3 independent experiments is shown.

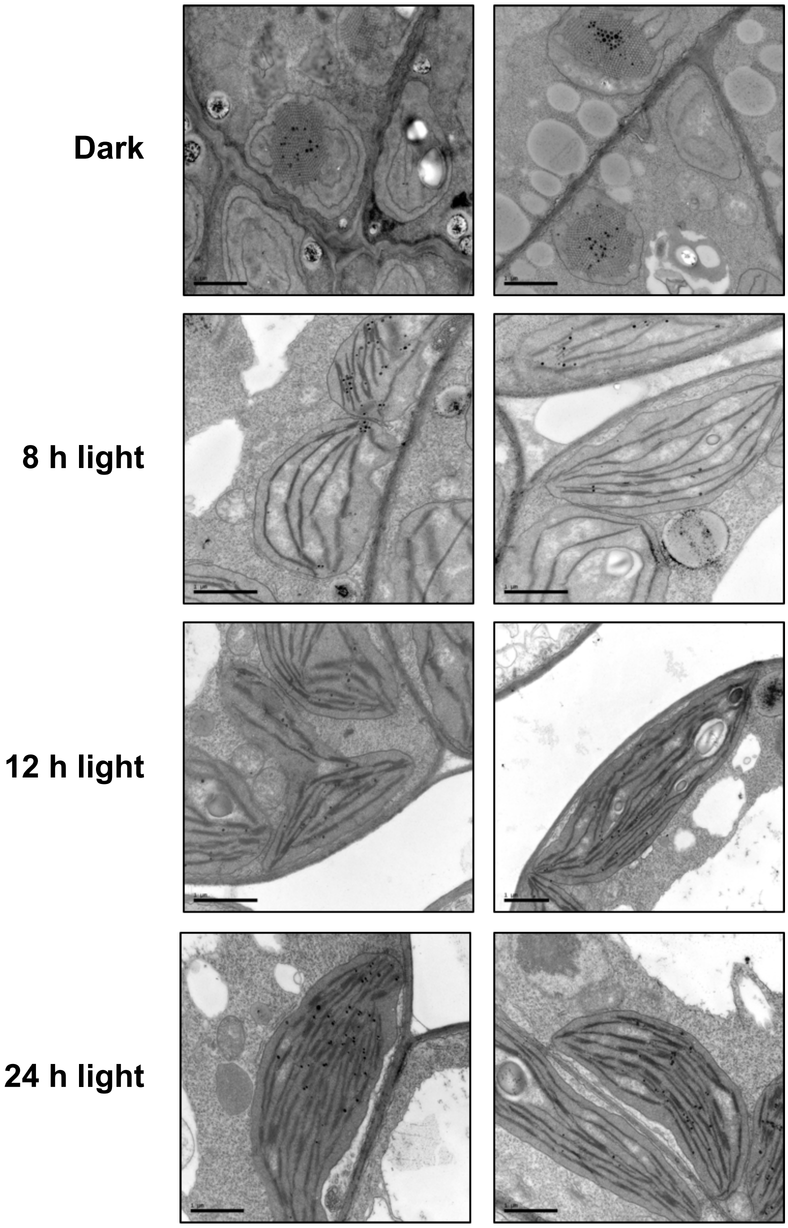

Supplementary Figure 6. Chloroplast and photosynthetic machinery development during de-etiolation.

Transmission electron micrographs of chloroplast from cotyledons of de-etiolated Arabidopsis WT seedlings in dark, and after 8, 12, and 24 h light exposure. Bars = 1μm.

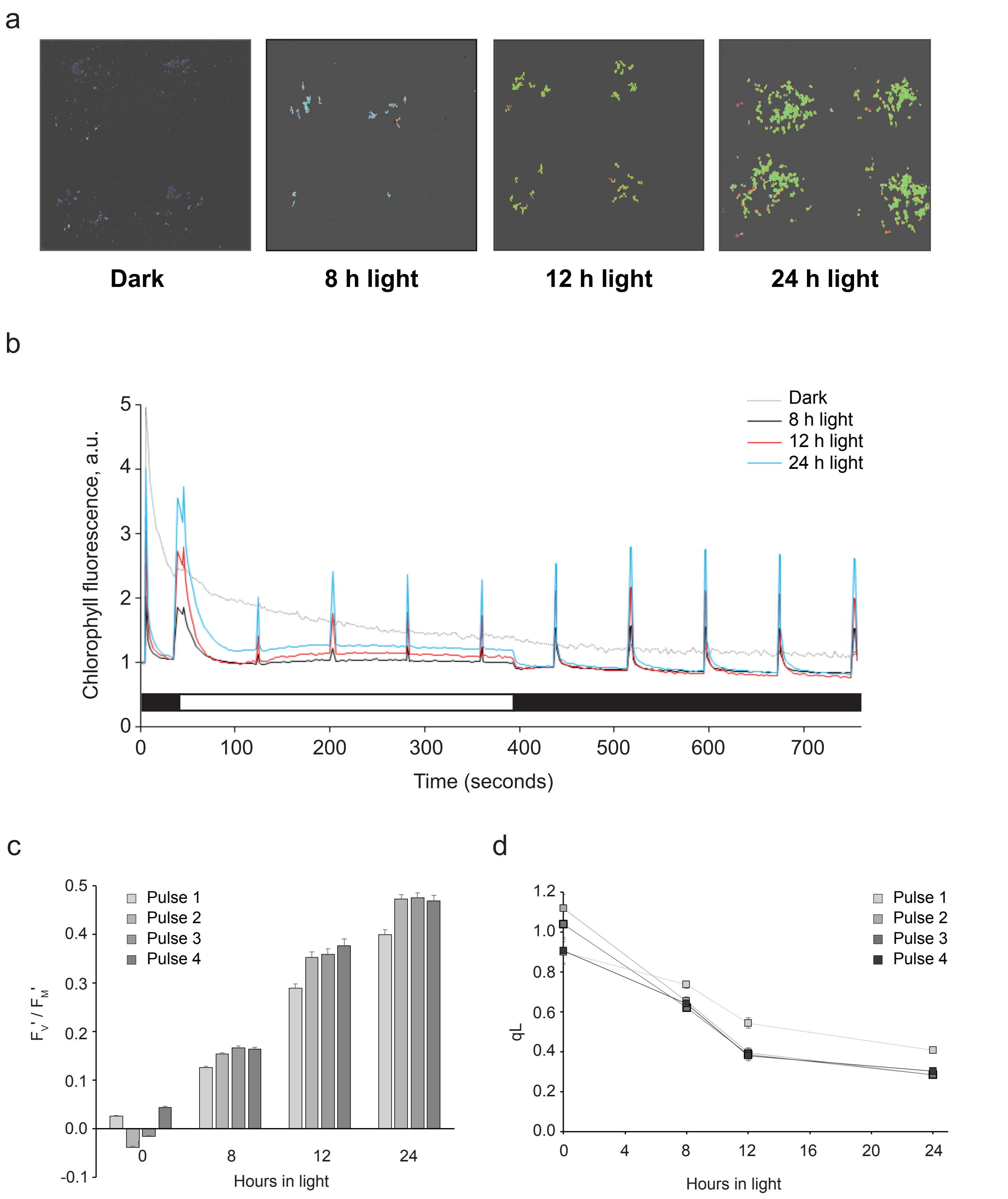

Supplementary Figure 7. Photosynthetic activity of Arabidopsis Col-0 seedlings during de-etiolation. (**a**) Raw images of chlorophyll *a* fluorescence of Arabidopsis seedlings measured after 0 (dark control), 8, 12 and 24 h in the light. (**b**) Kinetics of chlorophyll *a* fluorescence in seedlings after 0 (dotted black line), 8 (black), 12 (red) and 24 (blue) hours in the light. Application of white actinic light is indicated by the white bar below the traces, and dark periods by the black bars. The average kinetic traces from at least 20 seedlings are shown normalised to the same initial fluorescence. (**c**) Quantification of photochemical efficiency of PS II centres in the light-adapted state (F_V'/F_M'), derived from the fluorescence induction data shown in (b). Data shown are the mean ± s.e.m. of fluorescence derived from the first four saturating pulses following 60 s actinic illumination for at least 20 individual seedlings on two independent plates. (**d**) Quantification of open PS II centres (qL parameter; determined by $qL = [(F_M' - F)/ (F_M' - F_O')] * (F_O'/F)$) from the same data shown in (b).

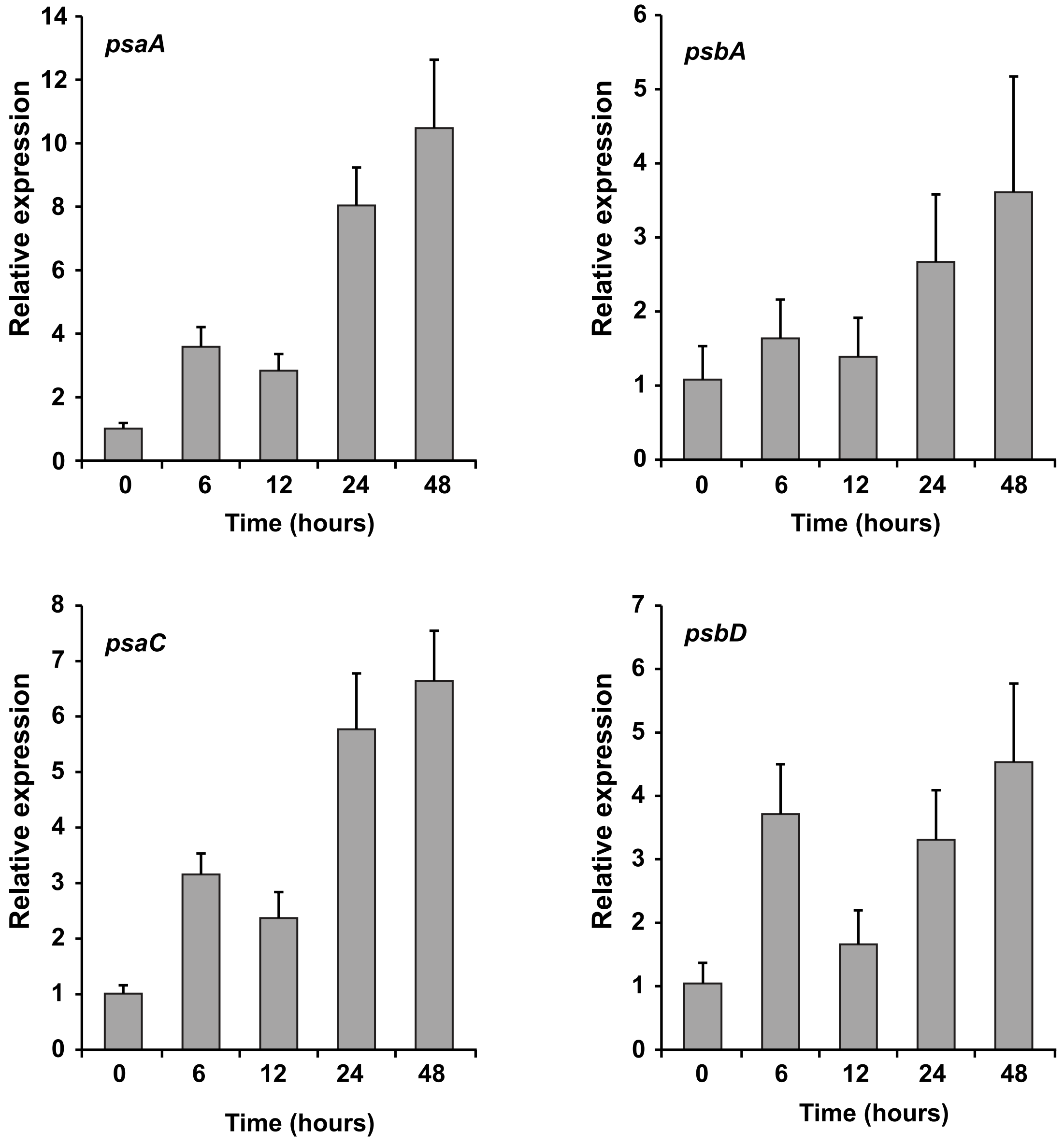

Supplementary Figure 8. Expression of plastid-encoded photosynthetic genes during de-etiolation.

Relative expression levels of PEP transcribed *psaA, psbA, psaC* and *psbD* genes in de-etiolated Arabidopsis WT seedlings at different times after exposure to light. Gene expression was normalized to *AT4G36800* and related to the amount present at time 0. Data represents the mean ± s.d. of at least three independent biological replicates.

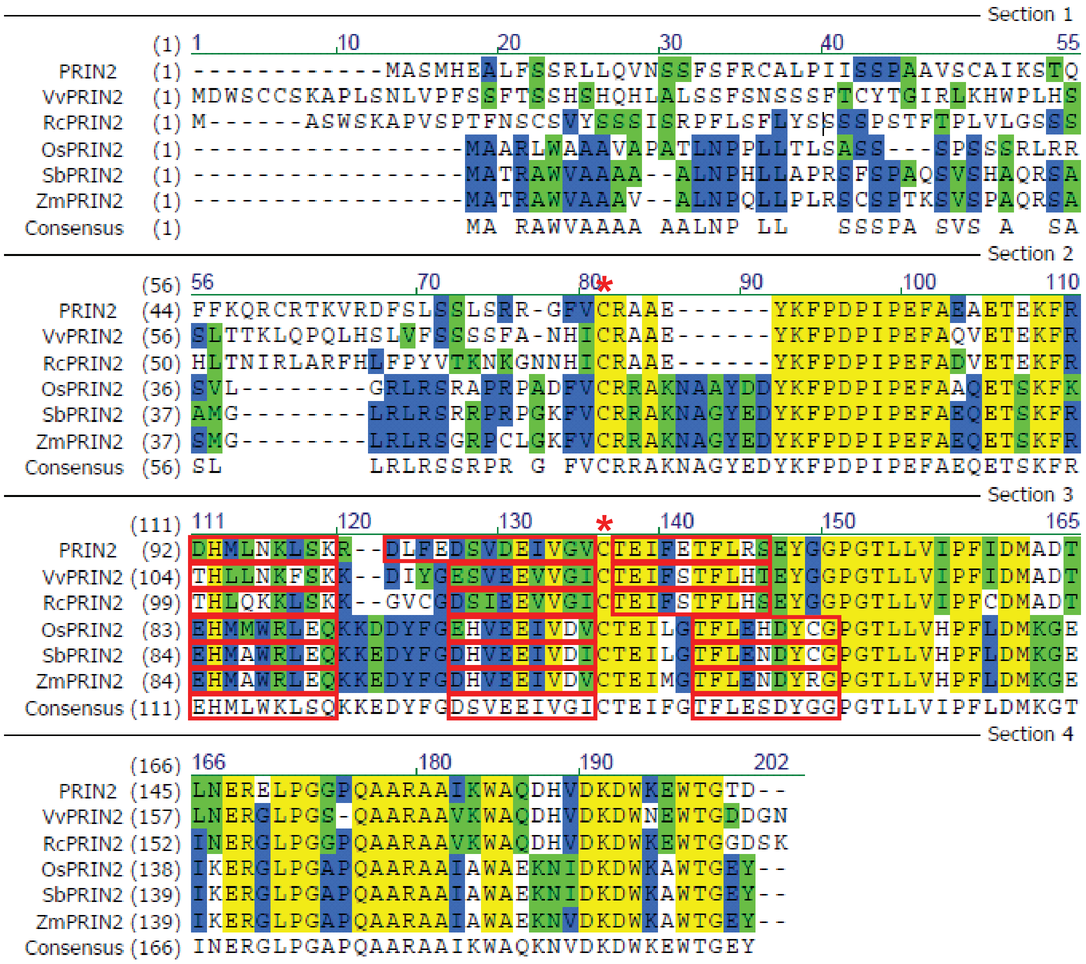

Supplementary Figure 9. Transactivation Domain.

Prediction of nine amino acids transactivation domain, 9aaTAD. Red boxes mark the residues predicted to form a 9aaTAD domain. The prediction was performed using the less stringent pattern in http://www.med.muni.cz/9aaTAD/. When a more stringent pattern was used the transactivator domain is only found in the sequences of the dicotyledonous plants, 2 in Arabidopsis, and 1 in *Vitis vinifera* and *Ricinus communis*. The amino acid sequences of PRIN2 proteins from *Arabidopsis thaliana*, *Vitis vinifera* (Vv), *Ricinus communis* (Rc), *Oryza sativa* (Os), *Sorghum bicolor* (Sc) and *Zea mays* (Zm) were aligned and manually adjusted. Identical residues are shadowed in yellow, 50% identical residues in blue, and similar residues in green. Red asterisks mark the conserved Cysteines.

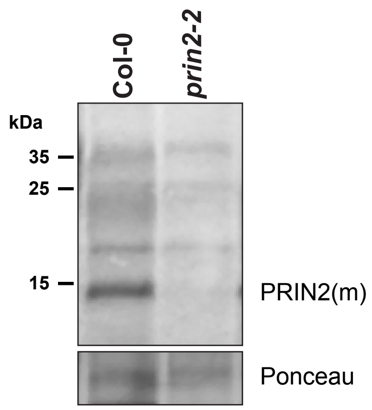

Supplementary Figure 10. Validation of anti-PRIN2 antibody.
Anti-PRIN2 antibody was validated using *prin2-2* mutant. Immunoblot analysis of PRIN2 with 70 μg of protein from isolated chloroplast from WT and *prin2-2*. Proteins were subjected to SDS-PAGE (15% polyacrylamide) under highly reducing conditions (100 mM DTT) and blotted.

Figure 4a

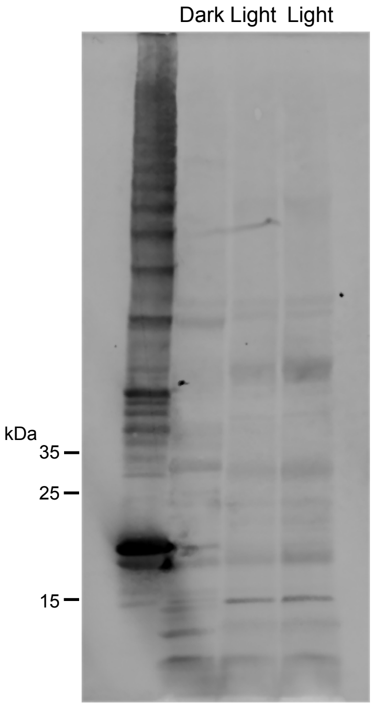

Figure 4b

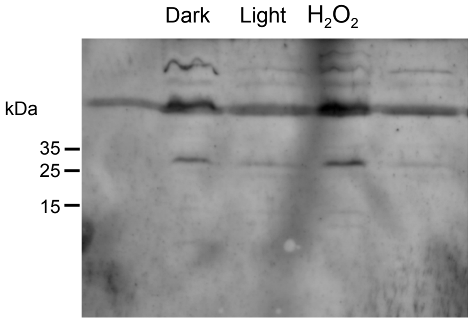

Figure 4b | Figure 4e

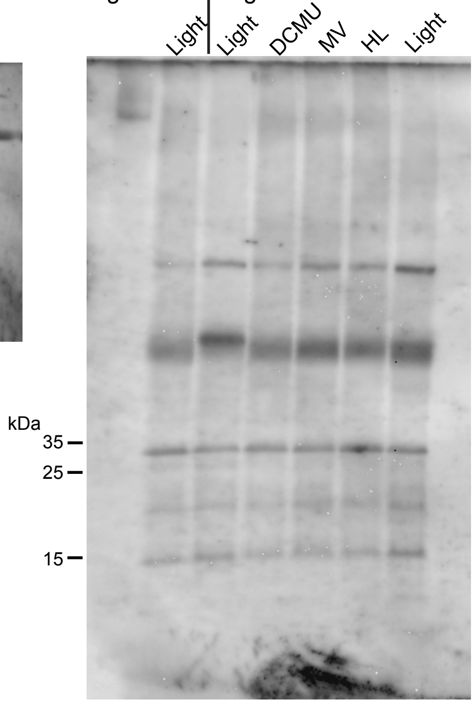

Figure S4b

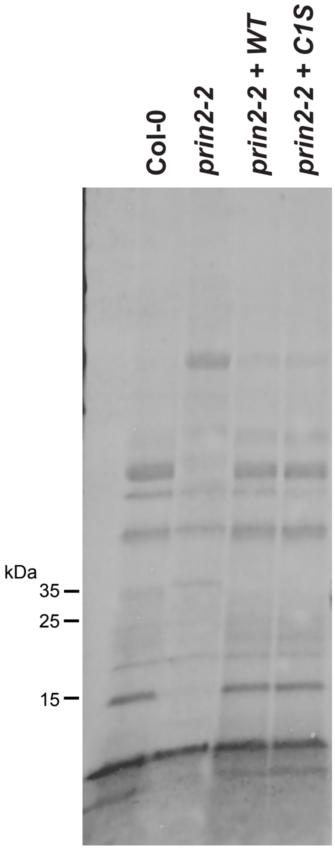

Figure S4b

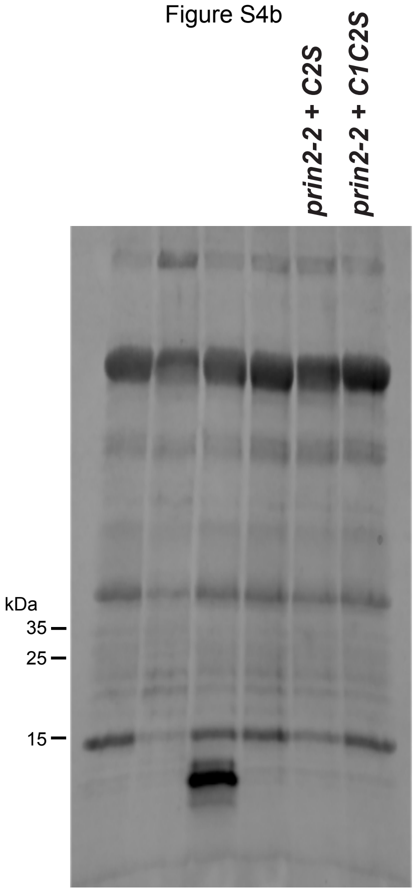

Figure S4c

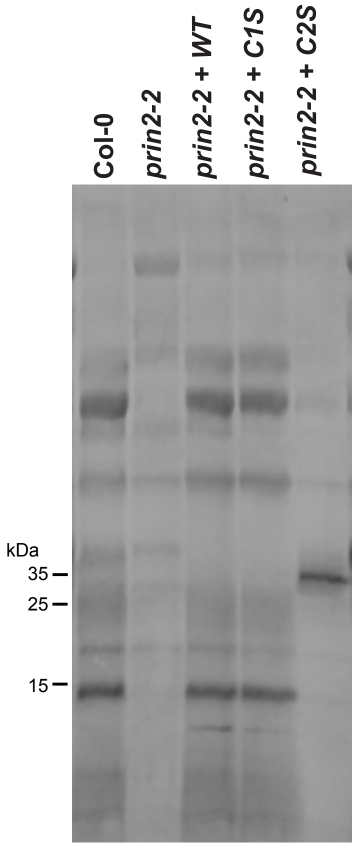

Supplementary Figure 11. Uncropped versions of blots.

Supplemental Table 1. Oligonucleotide sequences of primers used in this study.

Primer name	Sequence 5' to 3'
**Cloning and direct mutagenesis**	
PRIN2_CDS_GW_Fw	GGGGACAAGTTTGTACAAAAAAGCAGGCTATGGCTTCAATGCACGAAGCTC
PRIN2_CDS_GW_Rev	GGGGACCACTTTGTACAAGAAAGCTGGGTCTAATCAGTGCCGGTCCATTCC
68 Cys-Ser Rev Primer	CAGCAGCTCTGCTAACGAACCCT
68 Cys-Ser Fw Primer	AGGGTTCGTTAGCAGAGCTGCTG
prin2 c2.s Rev	TTACCTCAGTGCTGACTCCAACA
prin2 c2.s fw	TGTTGGAGTCAGCACTGAGGTAA
AttB1 PRIN2 endo Fw	GGGGACAAGTTTGTACAAAAAAGCAGGCTTCGGCATCACAGGCACACAGCCT
AttB2 PRIN2 Rev	GGGGACCACTTTGTACAAGAAAGCTGGGTGATCAGTGCCGGTCCATTCCTT
**qRT-PCR**	
Prin2 qPCR Fw	CCGAAGCTGAGACTGAGAAA
Prin2 qPCR Rev	GGTCCTCCATACTCACTGC
LHCB1.1 Fw	CGGAAAGTGAGCCAAGTTCT
LHCB1.1 Rev	TGAAAGTCTCTACCATCCACCA
LHCB2.4 Fw	GCCATCCAACGATCTCCTC
LHCB2.4 Rev	TGGTCCGTACCAGATGCTC
At4g36800 fw	CTGTTCACGGAACCCAATTC
At4g36800 rev	GGAAAAAGGTCTGACCGACA
PsaA_F	ACTACCACTTGGATCTGGAAC
PsaA_R	GTATTTCCATGGTGCTCGTTT
PsbA F	ATACAACGGCGGTCCTTATG
PsbA R	AGCAATCCAAGGACGCATAC
PsaC F	ATAGGATGTACTCAATGTGTCC
PsaC R	ATCTCTTACAACCAACACAGTC
PsbD F	TCATGGTATACTCATGGATTGG
PsbD R	GACCACCTAATTGACACCAACG
**EMSA**	
psaA-188-F	ATGACATATCCATAGGGTGCTC
psaA-188-R	AATAAGCATTTATTGAAATAGGA
bs-18N	CGCGAATTCGGATCCAAGC$(N)_{18}$CGTTGTCGACTC-GAGTCGA
rs-1	TCGACTCGAGTCGACAACG
pYCF1_F	CCATTCATTTAATATCCCTTTGGTGTCATTGACATAAGAGAT
pYCF1_R	ATCTCTTATGTCAATGACACCAAAGGGATATTAAATGAATGG
pRpoB_F	TATGGTATGCAATCGAATTGGAATATGTAATATCATAGGTGAAAATGAAATTAC
pRpoB_R	GTAATTTCATTTTCACCTATGATATTACATATTCCAATTCGATTGCATACCATA
PclpP_F	CGAAACCCCAATTTTACGTTTCCACATCAAAGTGAAATAGAGAA
PclpP_R	TTCTCTATTTCACTTTGATGTGGAAACGTAAAATTGGGGTTTCG
PpsaA region U_F	TTTTTTAATTATAAAAAAGGGTCCGTTGAGCACCCTATCCAT
PpsaA region U_R	ATCCATAGGGTGCTCAACGGACCCTTTTTTATAATTAAAAAA
psbA_197 F	GAAACAGTATAACATGACTTAT
psbA_197 R	ACCCACTACGGATCGTATTCAA
**Cloning for Co-IP**	
PRIN2 F	AAGCCATGGCATATGGCTTCAATGCACGAAGCT
PRIN2 R	AACCCATGGTTTCCTTCCAGTCCTTGTCTAC
TRXz F	ATCCGAGCTCATGGCTCTTGTTCAATCCAGA
TRXz R	CCTACCGCGGTGCATCTCGTTGTCAATGATA
**Cloning of recombinant proteins**	
PRIN2-Fw	GCTTCCATGGGCTCCCGGAGAGGGTTCGTT
PRIN2-Rev	GCTGTGTACCTAATCAGTGCCGGTCCAT
TRXz-trunc-Fw	GCTTCCATGGGCTTGTCAGCTCAAGAACTTCAGGAAC
TRXz-Rev	GCTTGGTACCTTACATCTCGTTGTCAATGATATCG

Supplementary methods

Transmission electron microscopy

Arabidopsis WT de-etiolated seedlings were fixed with 2.5% glutaraldehyde in 0.1 M cacodylate buffer overnight at 4°C, and washed 3 times in the buffer. The samples were post-fixed with 1% osmium tetroxide for 1 hour and washed with distilled water. Samples were dehydrated with 50, 70, 95 and 100% ethanol and embedded in Spurr´s resin. Sections were made with a diatome diamond knife, collected on copper grids, and treated with 5% uranyl acetate in water for 20 min followed by Sato´s lead staining for 5 min. Sections were examined in a JEOL 1230 Transmission Electron Microscope, and images were captured using Gatan MSC 600CW CCD camera.

Measurements of photosynthetic activity in Arabidopsis cell culture

Photosynthetic activity *in vivo* in the cell culture was measured using the chlorophyll *a* fluorescence function of a DUAL-PAM-100 fluorometer (Heinz Walz GmbH, Effeltrich, Germany). Briefly, cells were dark-adapted for 25 min on a shaker (~125 rpm) and 2 mL cells were transferred to a stirred cuvette for the analysis. F_V/F_M was determined using a saturating, single-turnover flash (3000 µmol photons m^{-2} s^{-1}, 600 ms) before application of a 100 µmol photons m^{-2} s^{-1} red actinic light and measurement of slow fluorescence kinetics.

Measurements of photosynthetic activity in Arabidopsis Col-0 seedlings during de-etiolation

Photosynthetic activity (PS II) was assessed by measuring chlorophyll *a* fluorescence with a Handy FluorCam (PSI Instruments, Brno, Czech Republic). De-etiolation was induced in constant white light of 150 µmol photons m^{-2} s^{-1} intensity at 22°C, and chlorophyll fluorescence measured using a PAM protocol. Seedlings were dark-adapted for 15 min before initial fluorescence (F_O) was measured using four red LED measuring pulses. Maximal fluorescence (F_M) was then measured following a saturating super pulse of 1000 µmol photons m^{-2} s^{-1} intensity for 800 ms. The PAM protocol measured the fluorescence during exposure to actinic white light of 100 µmol photons m^{-2} s^{-1} intensity (provided by LEDs) for 6 min, and maximal fluorescence in the light (F_M’) was measured every 60 s using super pulses. The actinic light was then switched off and fluorescence measured in the dark for another 6 min. Data was processed using the FluorCam 7 software.

Statistics

The graphs and the statistical analysis in Fig. 6 were done in Excel. No inclusion/exclusion criteria were used. No randomization was used to determine location or process of the data. The experiments and data analysis were performed without blinding. The replicates for experiments with seedlings were done with a pool of minimum 20 seedlings. All the samples were growth and collected in similar conditions.

Supplementary references

1. Okuda, K. *et al.* The pentatricopeptide repeat protein OTP82 is required for RNA editing of plastid *ndhB* and *ndhG* transcripts. *Plant J.* **61,** 339–349 (2010).
