# Supplementary material for: Redox regulation of PEP activity during seedling establishment in Arabidopsis thaliana
Source: Nat Commun. 2018 Jan 3;9:50. doi: 10.1038/s41467-017-02468-2 (PMC5752674; doi:10.1038/s41467-017-02468-2)
Supplement: Supplementary file 2 — Description of Additional Supplementary Files [file 41467_2017_2468_MOESM2_ESM.pdf]

Description of Supplementary Files

File Name: Supplementary Data 1
Description: The top ranked genes co-expressed with PRIN2.
